# Recent advances in fluorescent probes of peroxynitrite: Structural, strategies and biological applications

**DOI:** 10.7150/thno.80529

**Published:** 2023-03-13

**Authors:** Jiarao Sun, Xiaoli Cao, Wenjuan Lu, Yongchun Wei, Lingxiu Kong, Wei Chen, Xintian Shao, Yanfeng Wang

**Affiliations:** 1 Institute of Materia Medica, Shandong First Medical University & Shandong Academy of Medical Sciences, Jinan 250000, Shandong Province, China; 2 Jinan Municipal Center for Disease Control and Prevention, Jinan 250021, Shandong, China; 3 School of Life Sciences, Medical Science and Technology Innovation Center, Shandong First Medical University & Shandong Academy of Medical Sciences, Jinan 250062, Shandong, China

**Keywords:** peroxynitrite, fluorescent probes, nanoparticles, response mechanisms, bio-imaging

## Abstract

Peroxynitrite (ONOO^-^), owing to its high oxidative and nitrating stress, is associated with several physiological processes in addition to various pathological processes, including those related to neurodegenerative diseases and cancer. Detection of ONOO^-^ at the cellular level is of great significance to understand its pathogenesis. To this end, a variety of fluorescent probes based on small molecules and nanoparticles (NPs) have been engineered and applied as excellent tools for imaging of ONOO^-^ in cells as well as in their diverse biological applications. In this review, we highlight representative cases of fluorescent probes based on recognition mechanism and emphasize their response type (ratiometric, two-photon, long-wavelength/near-infrared, and targeting) in ONOO^-^ detection in the last five years. We further discuss their design strategy, sensing mechanism, and application in bio-imaging and describe NP-based probes according to diverse nanoplatforms.

## Introduction

**Figure 1:**
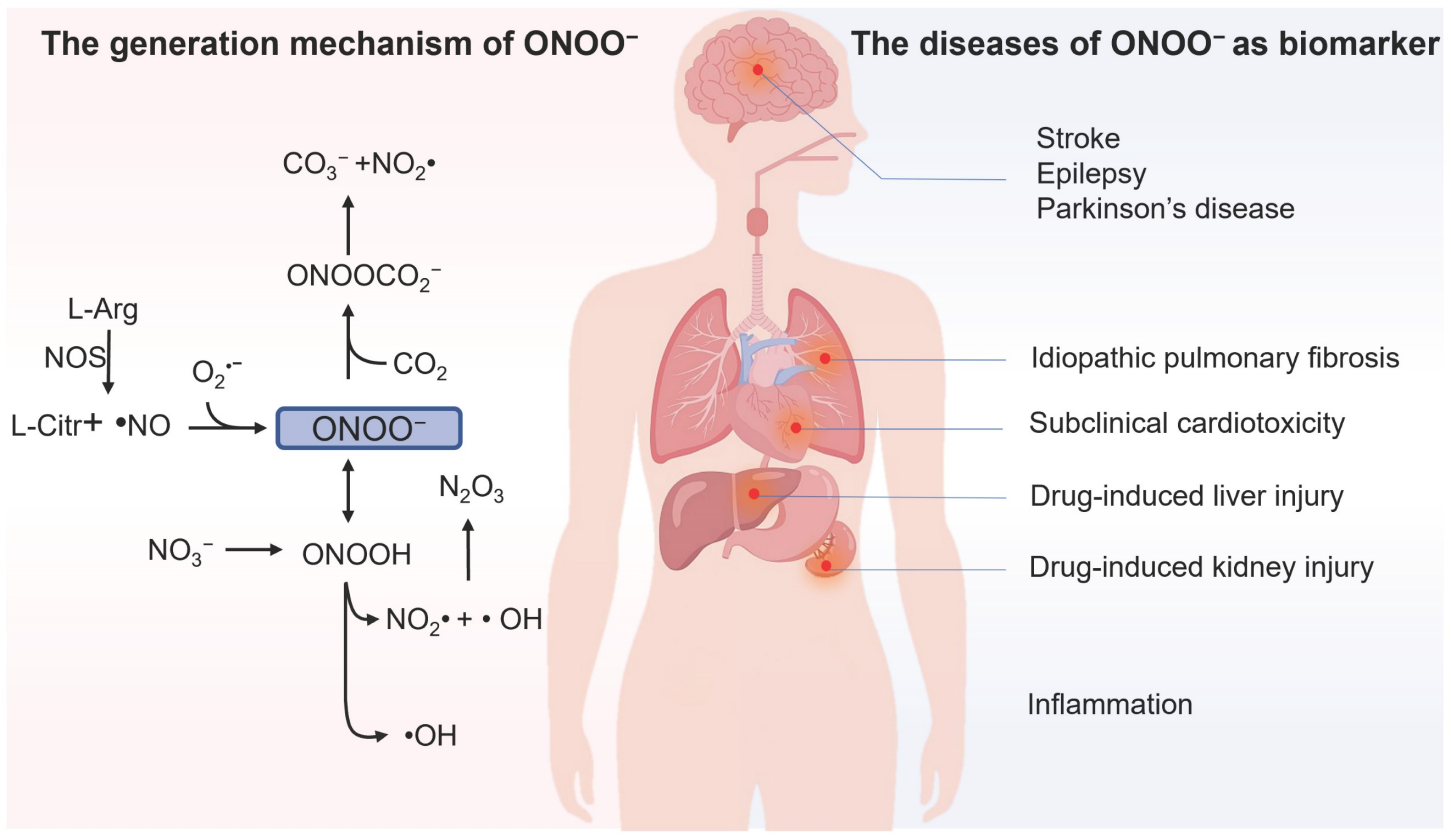
Illustration of the generation mechanism / metabolism of ONOO^-^ and the diseases of ONOO^-^ as biomarker.

Intracellular reactive nitrogen species (RNS) are a class of substances primarily originated from nitric oxide (NO) through electron transfer reactions, including nitric oxide free radicals (NO*•*), nitrogen dioxide free radicals (NO_2_*•*), hyponitric acid (HNO), nitrite ion (NO_2_^-^), and peroxynitrite (ONOO^-^) 1, 2. Among them, ONOO^-^ attracts special attention as its level changes are closely associated with various physiological and pathological processes in biological systems. In 1990, ONOO^-^ was first reported to be an endogenous oxidant 3. As depicted in Figure 1, ONOO^-^ is generated by NO and superoxide anion radical (O_2_**^•^**^-^) through a radical coupling reaction. ONOO^-^ as a nucleophile can react with carbon dioxide (CO_2_) to generate an unstable intermediate (ONOOCO_2_^-^). This intermediate later decomposes and produces carbonate (CO_3_^2-^), which that can result in apoptosis and programmed cell death. Furthermore, the protonated form of ONOO^-^ (ONOOH, pKa = 6.8) can decompose into *•*OH and NO_2_*•*, which are considered deleterious in most cases owing to their high reactivity. Thus, ONOO^-^ acts as a signaling molecule *in vivo* for a number of pathways.

Although ONOO^-^ often plays protective roles in cells and organisms, such as the bactericidal effect, it has been reported to be deleterious in most cases owing to its high reactivity 4. It can be involved in cell death by oxidation of biological targets such as lipids 5, proteins 6 and DNA 7. Abnormal ONOO^-^ levels are closely linked to the diversity of human diseases; for instance, ONOO^-^ was found as a direct indicator of acute liver injury (ALI) associated with overdosage of analgesic/antipyretic drugs, as they undergo enzymatic biotransformation in the liver to generate ONOO^-^ through a cascade of oxidation reactions 8. Epilepsy is a chronic neurodegenerative disease, and its pathological progression is closely associated with ONOO^-^ 9. Furthermore, the outburst of ONOO^-^ takes place in the inflammatory processes, and the overproduction of ONOO^-^ was found to be proportional to the developing progression of inflammation 10. Moreover, abnormal ONOO^-^ levels are closely linked to the diversity of human diseases, such as neurodegenerative disorders 11, stroke 12, and drug-caused acute kidney injury (AKI) 13, among others. Therefore, developing highly sensitive and selective detecting tools for ONOO^-^ in biosystems is of significant importance.

Compared with traditional colorimetric analysis and electrochemical analysis, fluorescence analysis has been acknowledged by the scientific community as a more efficacious detection tool in living systems owing to its strong selectivity, high sensitivity, operational and structural modification simplicity, and visualization. 14-17 With the development of fluorescence analysis technology, the number of fluorescent probes used to detect ONOO^-^ gradually increased. Due to the important role in ONOO^-^* in vivo*, many ONOO^-^ fluorescent probes have been developed and applied in living cells. 18, 19 In terms of imaging technology, super-resolution fluorescence microscopy techniques successfully overcome the limitation of traditional optical microscopes, which cannot obtain spatial resolution below 200 nm. Furthermore, the former can be used for real-time observation of changes in the sub-organelles before and after response with ONOO^-^. This review is anticipated to make tremendous contributions in further understanding the role of ONOO^-^ in the biological and pathological processes.

**Figure 2:**
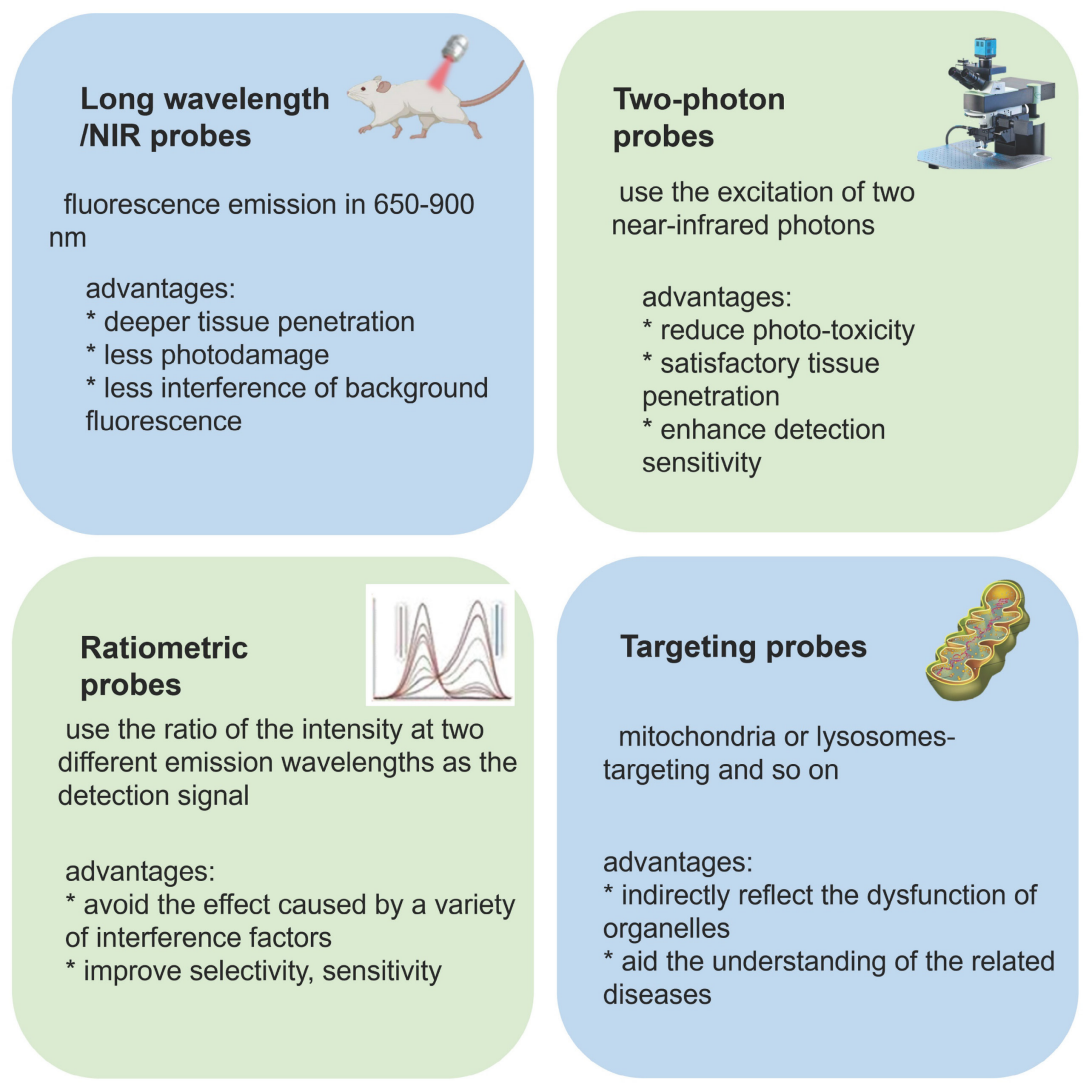
Different features of probes and their advantages.

Fluorescent probes for ONOO^-^ detection that have been applied in bio-imaging in the past five years are reported herein. We discuss small molecule-based fluorescent probes, in regard to recognition mechanism, response type (ratiometric, two-photon, long-wavelength/near-infrared [NIR], and targeting) (Figure 2), design strategy, sensing mechanism and application in biological imaging. We describe nanoparticle (NP)-based probes according to diverse nanoplatforms. Finally, we present our perspective on the development of new probes for the imaging of ONOO^-^ in living systems. We hope that this review is helpful to researchers interested in designing new probes for detecting ONOO^-^.

## Small-molecule-based fluorescent probes for detection of ONOO^-^

### Probes based on boronates (boronic acids/boronic esters)

**Figure 3:**
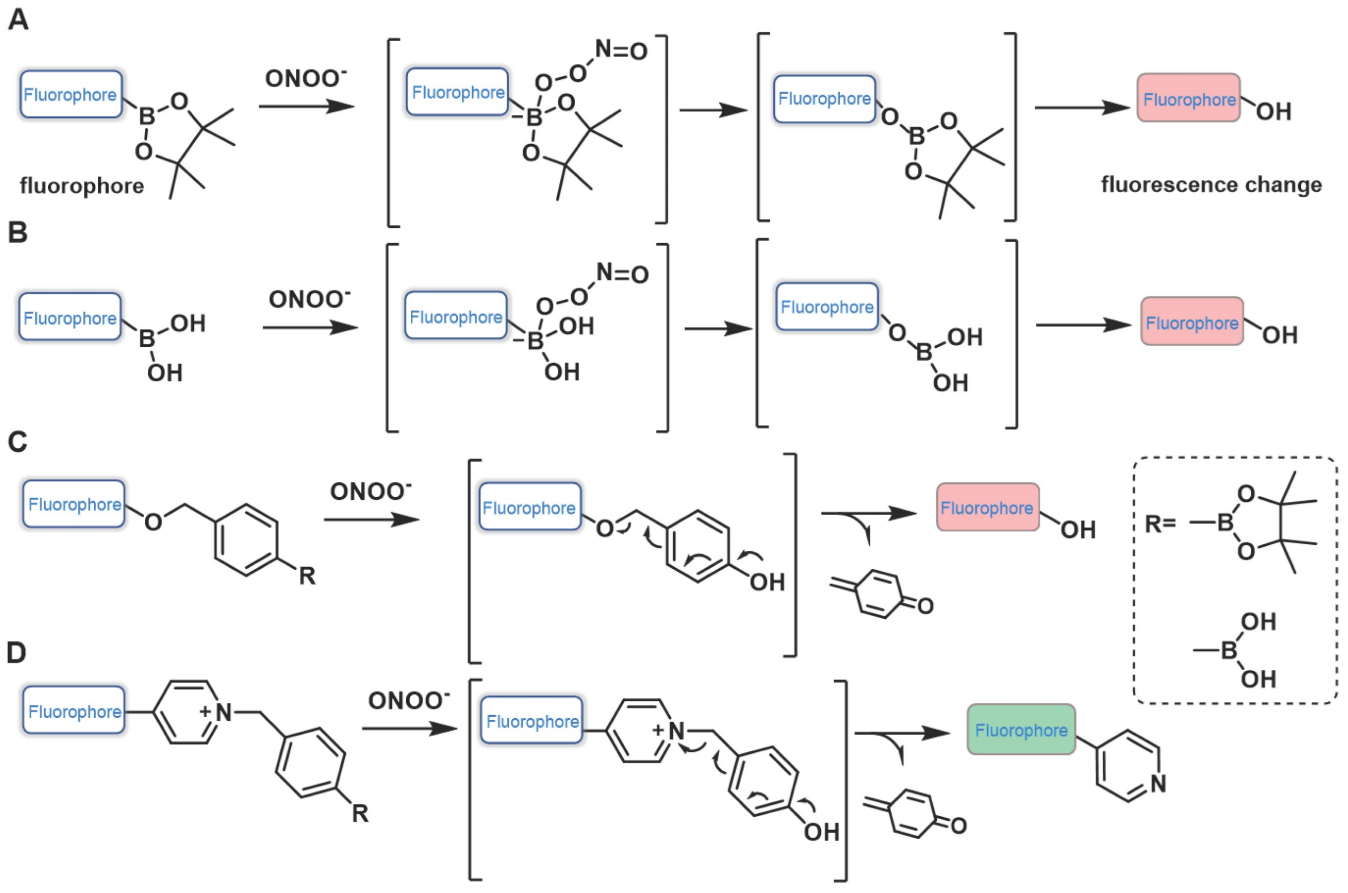
Design strategies based on boronates.

When fluorescent probes contain boronic acid or boronic ester moiety, they are generally believed to produce a specific response to hydrogen peroxide (H_2_O_2_). That is, the boronic acid or boronic ester group is generally considered to be a specific response site of H_2_O_2_. However, studies have demonstrated that boronic acid or boronic ester groups can react with ONOO^-^ millions of times faster than they react with H_2_O_2_ owing to the former's greater nucleophilicity _(_*k*_(ONOO_^-^ ~10^6^ M^-1^s^-1^, *k*_(H2O2)_ ~1 M^-1^s^-1^) 20. When designing the probe, in addition to directly connecting the boronate ester / boric acid group to the fluorophore, benzene or other linkers can be introduced between them (Figure 3). Fluorescent probes have been widely used for ONOO^-^ detection designed using this strategy. Moreover, we found that the fluorescent probes with boronic ester as the response site have shorter response time and higher selectivity when responding to ONOO^-^, which may be ascribed to the lower redox potential of borate ester 21, 22.

**Figure 4:**
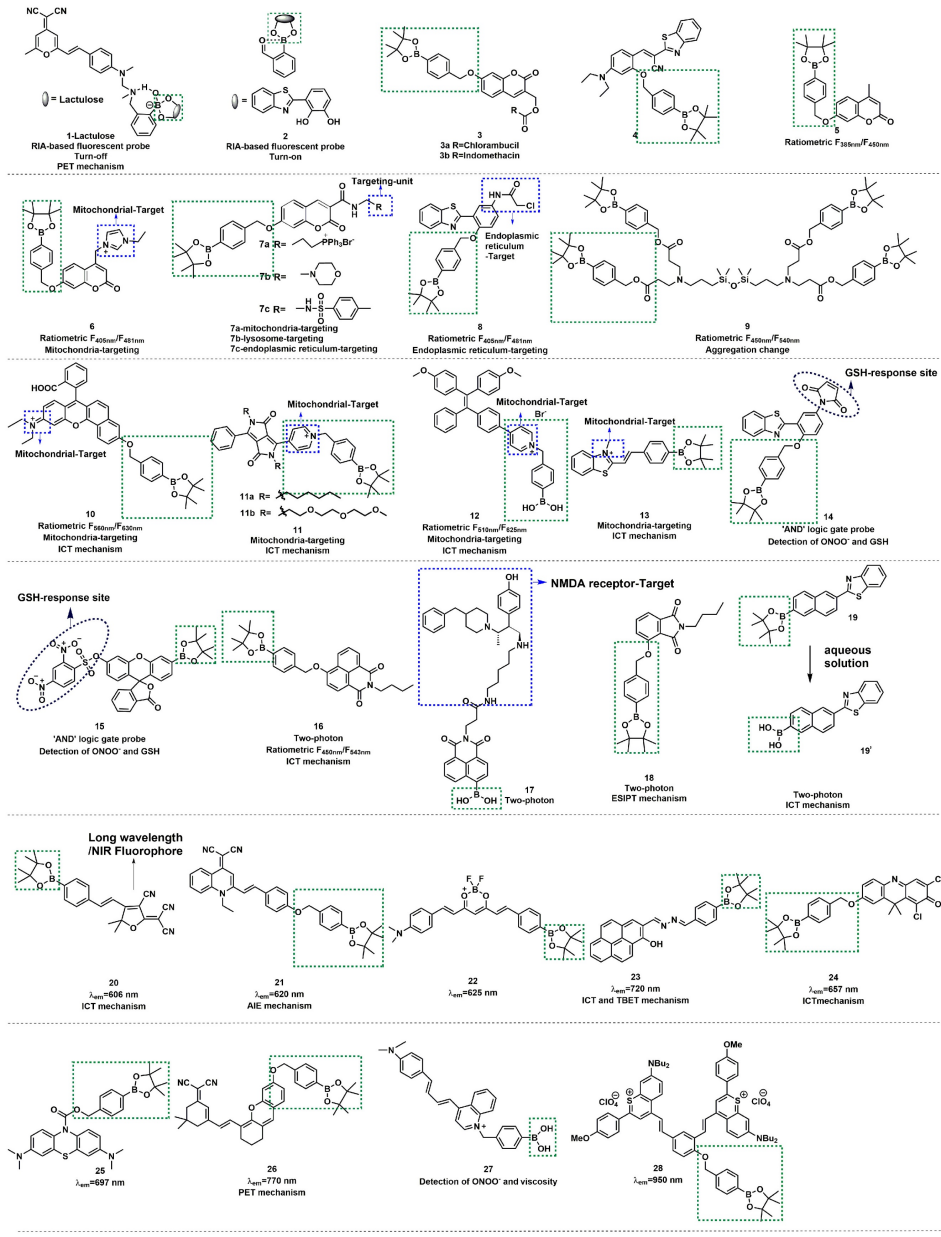
The structures of boronic acid / boronic ester-based fluorescent probes (**1**-**28**) for ONOO^-^ detection. The green boxes indicate the ONOO^-^ response unit, and the blue boxes indicate the targeting moiety.

In 2017, utilizing reaction-based indicator displacement assay (RIA) mechanism, Han *et al.* developed fluorescent probe **1** for detecting ONOO^-^ (Figure 4) 23. **1** was synthesized by introducing a boronic acid site into DCM (4-dicyanomethylene-2-methyl-6-[4-(dimethylamino) styryl]-4Hpyran), a laser dye. It had negligible fluorescence due to the photoinduced electron transfer (PET) effect. After binding with lactulose to form a complex, the PET effect was prohibited, and the fluorescence was restored. Upon addition of ONOO^-^, lactulose was dissociated from the complex, and the fluorescence was dramatically reduced owing to the recovery of the PET effect. The complex had better selectivity for ONOO^-^ than other reactive oxygen species (ROS) /RNS tested and demonstrated good imaging ability for exogenous ONOO^-^ in HepG-2 cells.

Similar to lactulose, 2-(2', 3' -bihydroxyphenyl)benzothiazole (BHBT) can provide two adjacent hydroxyl groups that react with boric acid to form a fluorescent borate ester. Xu* et al.* obtained a novel ONOO^-^ probe **2** by means of the self-assembly comprising BHBT and 2-formylphenyl-borbonic acid (Figure 4) 24. **2** was a typical “turn-on” type fluorescent probe, which was more suitable for biological imaging and overcame the disadvantage of the “turn-off” type fluorescent probe of being susceptible to autofluorescence interference. **2** has undergone integration, release, and recombination processes. After specific responses with ONOO^-^, the released product salicylaldehyde triggers cross-linking to fixed BHBT through hydrogen bond, enhancing the detection signal and improving the sensitivity. To explore the application of ONOO^-^ probes in the medical domain, James *et al.* prepared probes **3a** and **3b** (Figure 4) 25. In their study, coumarin was a fluorophore linker that separately conjugated cancer drugs chlorambucil and indomethacin with an ONOO^-^ trigger. The results demonstrated that **3a** and **3b** were non-fluorescent; however, the authors noted a strong increase in emission at 460 nm for both probes with the increasing concentrations of the biological oxidant ONOO^-^, which provided a potential platform for monitoring ONOO^-^-mediated drug release in cancer cell lines. With iminocoumarin-benzothiazole as a fluorophore, Wang *et al.* reported turn-on probe **4** (Figure 4) 26. The fluorescent intensity of **4** at 530 nm was found to have a good linear relationship with the concentration of ONOO^-^ in the range of 0-10 μM. Drug-induced hepatotoxicity represents critical challenge for safety in drug development. The production of ONOO^-^ has been proposed as an early sign in the progression of drug-induced hepatotoxicity. After successfully applying probe **4** for imaging ONOO^-^ in living cells, the studies to detect ONOO^-^ in drug-damaged liver tissues were conducted. Bright green fluorescence was obtained even when the depth was up to 80 μm in the liver tissues of drug-induced liver injury.

Ratiometric fluorescent probes function by using the ratio of two different emission intensities as detection signals, which can eliminate the interference of environment and instrument parameters, among other factors. Furthermore, owing to the high sensitivity and high selectivity of these probes, they have gained massive attention from the scientific community 27, 28. Wang* et al.* reported a ratiometric fluorescent probe **5** using 4-methylumbelliferone as a fluorophore (Figure 4) 28. The ratio of the emission intensities *F*_450 nm_/*F*_385 nm_ had a good linear relationship with the concentration ONOO^-^ in the range of 0-10 µM, and the limit of detection (LOD) was calculated to be 29.8 nM. **5** was successfully applied in the fluorescent imaging of endogenous and exogenous ONOO^-^ in living cells, zebrafish, and live tissues from a high-fat diet-induced obese mouse model. In 2022, Radosław* et al.* synthesized probe **6** with mitochondrial targeting function by introducing imidazolium into probe **5** (Figure 4) 29. Compared with **5**, the fluorescence emission peaks of **6** showed a certain degree of red shift (*F*_450 nm_/*F*_385 nm_ to *F*_481 nm_/*F*_405 nm_). Also based on this theory, using coumarin and 4-amino-2-(benzo[d]thiazol-2-yl) phenol (ABAH) as fluorophores, James *et al.* designed and synthesized probes **7a-c** and** 8** to detect ONOO^-^ by introducing different targetable groups (Figure 4) 30, 31.

Lin *et al.* devised a novel ratiometric probe **9** based on an unconventional fluorescent platform (Figure 4) 32. **9** was a novel four-armed siloxane containing a Si-O-Si bond as a bridge. Upon reaction with ONOO^-^, the hydrolysis of the boronic ester group changed the aggregation state of **9**, and the ratio of emission intensities (*F*_450 nm_/*F*_385 nm)_ had a good linear relationship with the concentration of ONOO^-^ in the range of 4-45 µM. In addition, **9** was used to visualize exogenous and endogenous ONOO^-^ in cells and zebrafish.

Ratiometric fluorescent probes with long-wavelength emission (λ_em_ >600 nm) have been attracted more attention from scholars because of their excellent superiorities of deeper tissue penetration, low tissue light damage, and less autofluorescence interference, in addition to the advantages of conventional ratiometric probes. Additionally, mounting evidence has demonstrated that the intramolecular charge transfer (ICT) mechanism is a preferred strategy for designing various ratiometric fluorescent probes. In 2018, Zhu* et al.* designed and synthesized probe **10** based on the ICT mechanism (Figure 4) 33. Their experimental results demonstrated that with the addition of ONOO^-^, the fluorescent emission of **10** was significantly red-shifted from 560 nm to 630 nm due to the change of the ICT effect. **10** showed outstanding selectivity, excellent sensitivity, fast response (< 5 s), and low LOD for ONOO^-^ (0.9 nM). Based on these advantages, **10** has been used for fluorescent imaging of endogenous ONOO^-^ in living cells.

**Figure 5:**
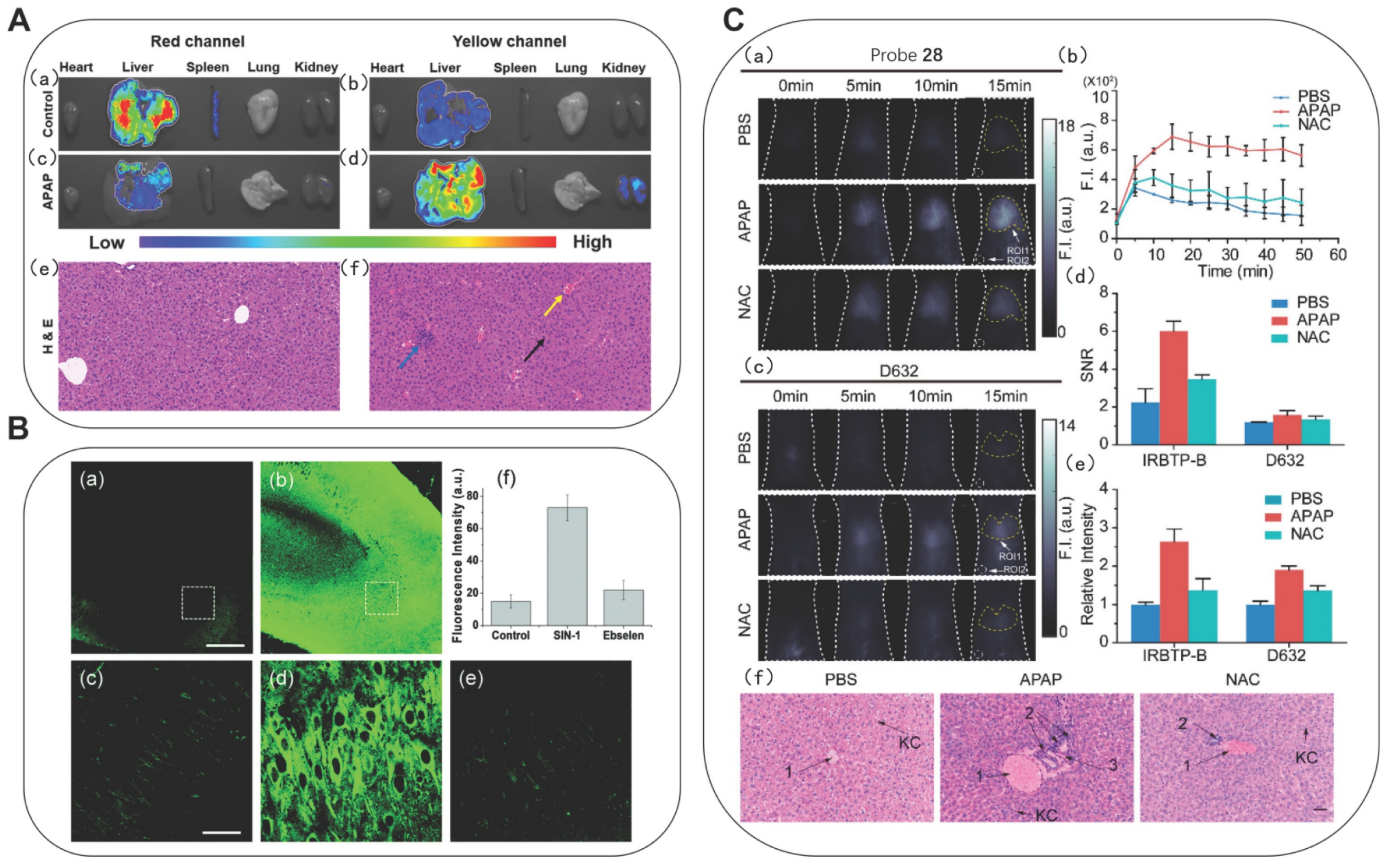
(A) Fluorescence of probe **11a** in organs from APAP-induced mice. (a, b) The mice of the control group; (c, d) APAP (300 mg/kg); H&E staining of liver tissues from the above mice. (e) Normal liver tissue. (f) Liver tissue of mice treated with APAP (300 mg/kg) for 12 h. Adapted from 34 with permission. (B) TPM rat hippocampal slice images acquired after incubation of 20 mM probe **18** for 1 h. (a) absence and (b) presence of 50 mM SIN-1. (c-e) Enlarged images show a white box part of panels a and b and were acquired (c) before and (d and e) after the addition of (d) SIN-1 (50 mM) for 20 min, (e) 150 mM ebselen with 50 mM SIN-1 for 40 min. (f) Average TPEF intensity in panels c-e. Adapted from 46 with permission. (C) Fluorescence images of endogenous ONOO^-^ in the livers of mice during an APAP-induced hepatotoxicity by probe **28** and D632. (a) *In vivo* imaging of livers of mice from probe **28** treated with various substances: PBS, APAP, and NAC + APAP. (b) Relative fluorescence intensity of livers of mice treated with various substances followed by probe** 28** over time. (c) In vivo imaging of livers of mice from D632 treated with various substances. (d) SNR values obtained for probe** 28** and D632 from different groups. (e) Relative intensity of livers of mice after injection of probe **28** and D632 in different groups. (f) Representative histology H&E of the livers of mice treated with various substances. Adapted from 56 with permission.

Also based on the ICT mechanism, Zhao* et al.* developed lipid-soluble probe **11a** and water-soluble probe **11b** (Figure 4) 34. They achieved excellent ratiometric detection for ONOO^-^ under physiological simulation conditions. Furthermore, the **11a** could originally target lysosomes. After successfully applying probe **11a** for imaging of ONOO^-^ in living cells, the authors further applied **11a** to the research of acetaminophen (APAP)-induced liver injury in mice. It is well-known that an overdose of APAP, a commonly household antipyretic and analgesic agent, is the leading cause of hepatotoxicity and even acute liver failure due to the overproduction of RNS. As shown in Figs. 5A(c) and 5A(d), the fluorescence signals of the mice in the experimental groups after administration were concentrated in the yellow channel. This was probably because excessive APAP induced liver damage in mice, leading to an up-regulation of ONOO^-^ in the liver. In the meantime, the fact that excessive APAP could induce liver damage in mice was proved via hematoxylin and eosin (H&E) staining. In contrast to the normal mice, the liver tissue of mice after administration exhibited obvious damage (Figure 5A(f)). **11a** could serve as a promising candidate to reveal the roles of ONOO^-^ in drug-induced liver injury and its repair. Zhang *et al.* synthesized a mitochondria-targeted AIEgen fluorescent probe **12** (Figure 4) 35. Probe **12** had an LOD as low as 0.45 nM.

The above studies indicated that ONOO^-^ fluorescent probes which could target different sub-organelles, are mainly composed of the following three parts: fluorophores, recognition site, and targetable unit.

Based on this, a large number of fluorescent probes for detecting ONOO^-^ and targeting sub-organelles such as mitochondria and lysosomes have been reported. The mitochondria membrane matrix has a strong negative potential, i.e., as low as -180 mV. Therefore, the most common method to deliver molecule probes to cellular mitochondria is to use positive charge groups, 36, 37 such as indolium moiety, quaternized pyridine and so on 38. Besides, fluorophores that possess lipophilic cations are also introduced to achieve mitochondria-targeting, such as cyanine dyes and rhodamine. The probes with these types of positively charged groups can travel through the plasma membrane and eventually accumulate in the mitochondria. Zhang* et al.* synthesized fluorescent probe **13**, containing a positively charged nitrogen heterocyclic, primarily distributed in the mitochondria (Figure 4) 39. They noted that the probe had a large red-shift of absorption peak accompanied by a distinct color change from colorless to orange after reacting with ONOO^-^, and the fluorescence intensity at 569 nm was linearly related with the concentration of ONOO^-^ (0-10 μM). The authors further applied **13** to monitor fluctuations of endogenous ONOO^-^ levels in living cells and possible cross-talk between H_2_S and ONOO^-^ in cells.

Fluorescent probe** 13** has revealed the interaction of two active substances in the living cells. It is a common phenomenon that the levels of multiple active substances simultaneously change when cells undergo oxidative stress. The molecules that can bind multiple analytes and convert the multiple binding events into measurable outputs are molecular logic gates. James* et al.* developed a series of fluorescein-based “AND” logic gates, which could detect ONOO^-^ and GSH or F^-^ in cells simultaneously. Using the aforementioned ABAH as an ideal ESIPT fluorophore, they developed probe **14** by modifying the hydroxyl and amino groups on it (Figure 4) 40. The maleimide group and benzyl boronic esters are typical GSH and ONOO^-^ responsive sites, respectively. Only a small fluorescence increased when ONOO^-^ or GSH alone was added in **14**. However, a significant fluorescence increase was observed following the addition of the second analyte, and the order of addition of ONOO^-^ and GSH did not affect the final fluorescence intensity. The authors further directed their attention toward the development of longer-wavelength probes for multiple-analytes. In this context, they synthesized probe **15** that could detect ONOO^-^ and GSH simultaneously (Figure 4) 41.

Two-photon imaging has the advantages of low photo-toxicity, satisfactory tissue penetration, and high detection sensitivity. 42, 43 Recently, two-photon bioimaging has attracted special interest of scientists. Naphthalimide is an outstanding ICT fluorophore with satisfactory two-photon properties. In 2019, Zhu* et al.* designed and synthesized the first ratiometric two-photon fluorescent probe **16** (Figure 4) 44, which was suitable for fluorescent imaging of ONOO^-^ in living RAW 264.7 macrophage cells using two-photon microscopy (TPM). Using the same fluorophore and an ifenprodil-like tail as target N-methyl-_D_-aspartate (NMDA) receptor, Yoon *et al.* designed probe **17** (Figure 4) 45, which represented high sensitivity and selectivity for ONOO^-^. They employed TPM imaging and demonstrated that **17** could be used to monitor ONOO^-^ levels near NMDA receptors in fresh rat hippocampal tissues.

James* et al.* prepared a two-photon fluorescent probe **18** using 4-hydroxyisoindoline-1,3-dione of an ESIPT-based fluorophore (Figure 4), which was applied in exogenous ONOO^-^ in fresh rat hippocampal slices up to a depth of 110 μm 46. The authors attempted to ascertain whether probe **18** could be used to detect ONOO^-^ in mice tissue. Using TPM, the obtained the images of a portion of **18**-labeled fresh rat hippocampal slices (Figure 5B). Their experiments at a higher magnification revealed the ONOO^-^ levels in the individual cells. In the same year, using 6-(1, 3-benzothiazol-2-yl) naphthalen-2-ol as a scaffold, Radosław *et al.* reported probe** 19** and discussed its detection of three inflammatory oxidants, H_2_O_2_, ONOO^-^, and HClO (Figure 4) 47. After responding to the above three oxidation reagents, the fluorescence spectrum of **19** exhibited similar changes, but peroxynitrite was the fastest oxidizing agent among them.

Because of the ability of longer excitation/emission wavelengths to allow deeper tissue penetration and mitigate photodamage to biological samples and background autofluorescence from proteins, therefore, the development of long-wavelength/NIR probes has been extensively investigated. Probes **20**-**27** were synthesized using different fluorophores (Figure 4) 48-55. With the addition of ONOO^-^, the boronic esters of these probes were oxidative to generate phenolic hydroxyl group, leading to a gradual increase in the peak intensity in the NIR region.

Probes with emission wavelengths in the visible range and the first NIR window (NIR-I, 650-900 nm) were limited by wavelength-dependent scattering and absorption behaviors, and the photon penetration depth was shallow in biological tissues. Probes with an emission wavelength in the NIR-II (900-1700 nm), were found to benefit from reduced self-absorption and scattering, and achieve deeper penetration depth and thus be better applied to biological tissue imaging. Zhang *et al.* synthesized probe **28** by introducing phenyl borate group into the benzothiopyrylium cyanines skeleton (Figure 4) 56. After reacting with ONOO^-^, the absorption peak was generated at 850 nm, accompanied by the reduction of the peak at 600 nm. Meanwhile, the emission peak appeared beyond 1000 nm. The ability of probe **28** to visualize the production of ONOO^-^ in a drug-induced hepatotoxicity model in nude mice was then assayed by comparison with the widely used commercial ONOO^-^ probe commercial D632 (Figure 5C). After the administration of APAP, the fluorescence intensities of the mice livers displayed gradual increments in a time-dependent manner, indicating the increased generation of ONOO^-^ after drug treatment. N-acetyl cysteine (NAC) was used as the antidote for APAP overdose. In contrast, no obvious fluorescent intensity augmentation was observed with time in the NAC pretreatment group, suggesting promising efficacy of NAC remediation. In comparison, signal-to-noise ratio (SNR) and relative fluorescent intensity were obtained from probe** 28**, indicating that probe** 28** is more sensitive than commercial D632 for in vivo imaging. The results of nude mice imaging showed that **28** could detect the changes in the ONOO^-^ level in the APAP-induced liver injury model.

### Probes based on C=C and C=N cleavage mechanism

**Figure 6:**
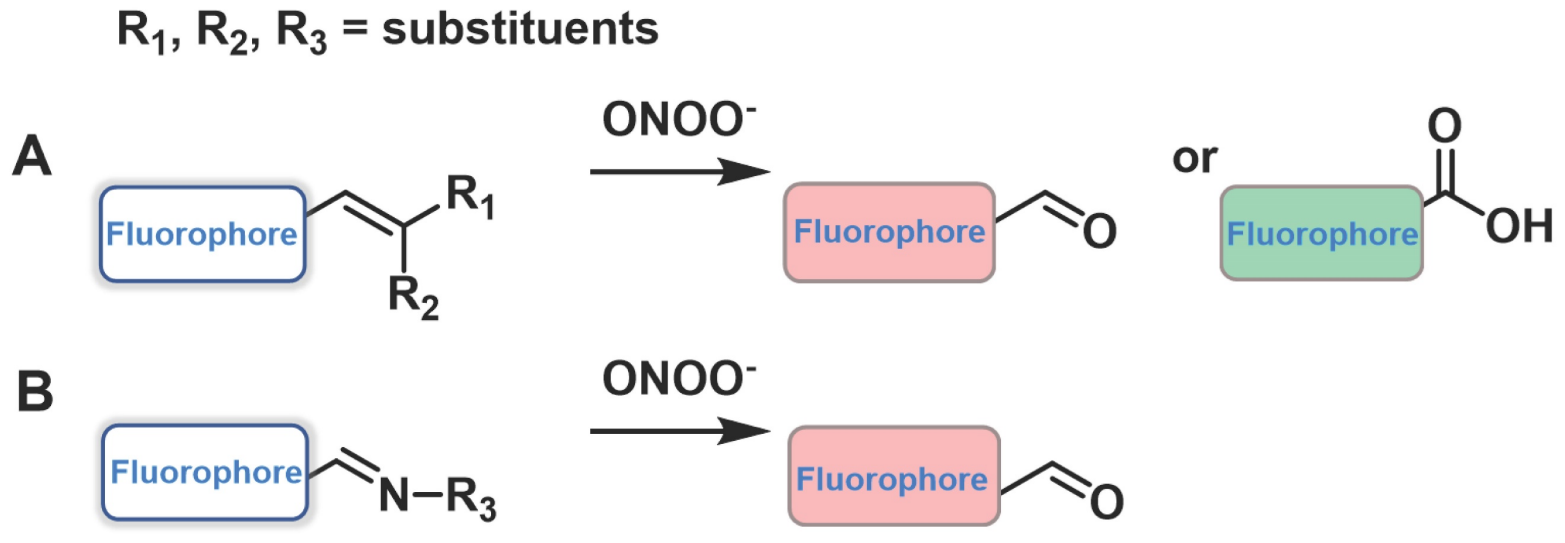
The design strategies based on C=C and C=N.

**Figure 7:**
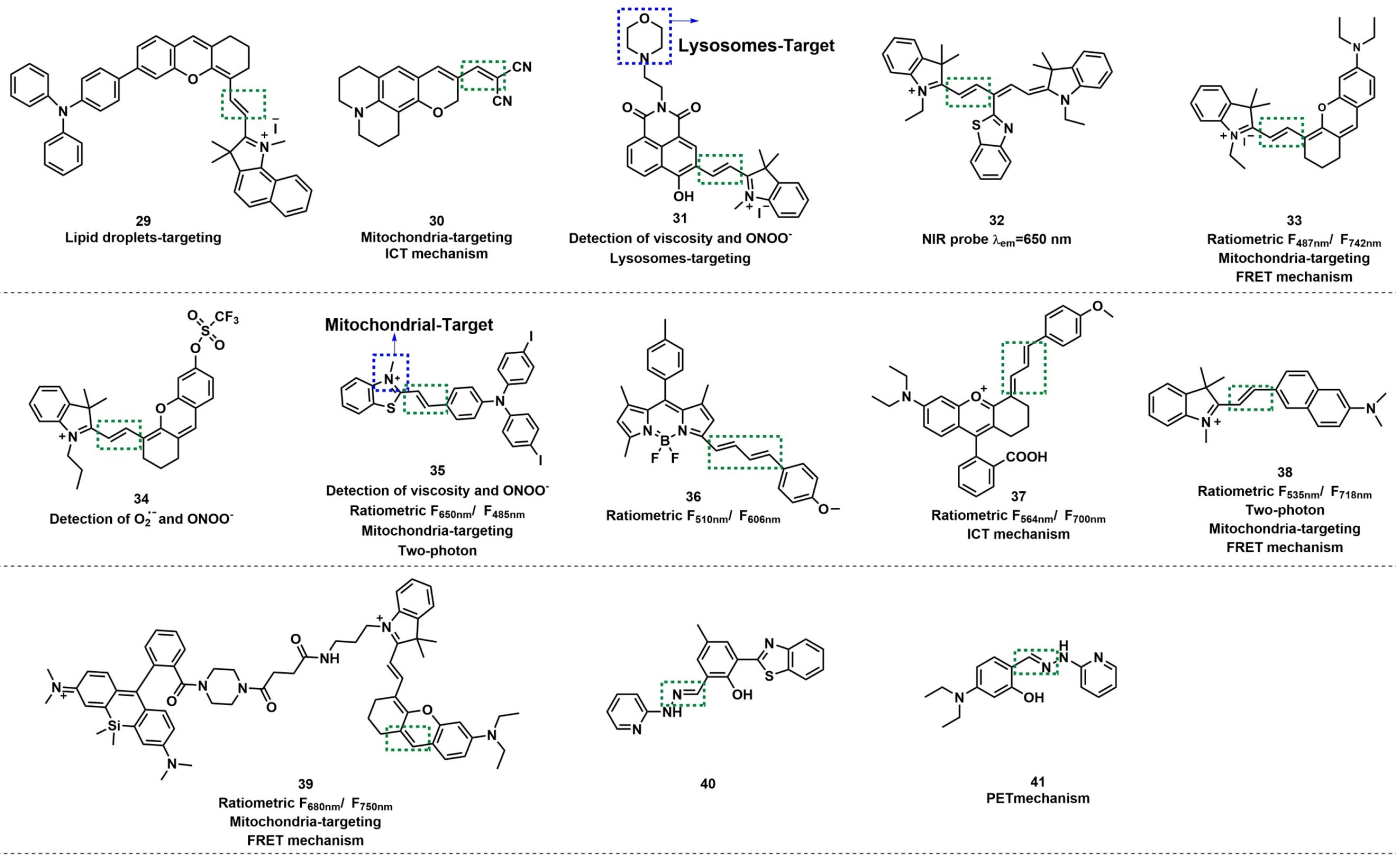
The structures of C=C/C=N-based fluorescent probes (**29**-**41**) for ONOO^-^ detection. The green boxes indicate the ONOO^-^ response unit, and the blue boxes indicate the targeting moiety.

Another sensing mechanism, the conjugated C=C double bonds and C=N double bonds could be cleaved through oxidation because of the strong oxidizing and nucleophilic ability of ONOO^-^, dramatically leading to a change in the fluorescent signal (Figure 6). Examples of this class of probes are presented in Figure 7, and the spectroscopic properties of the products formed are summarized in Table 2.

Based on a triphenylamine-benzoindolecyanine, Ye *et al.* developed a lipid droplet-targeted fluorescent probe **29** (Figure 7) 57. The C=C bond in this probe was broken after reaction with ONOO^-^, resulting in the destruction of the conjugate structure. The fluorescence was turned on to release benzoindolecyanine. With the increasing concentration of ONOO^-^, the absorption peak of **29** at 600-700 nm was gradually weakened; meanwhile, the fluorescence intensity at 456 nm was markedly enhanced and exhibited an excellent linear relationship with ONOO^-^ concentration in the range from 0 to 30 μM. Cell colocalization experiments with Nile red dye (lipid dye) indicated that **29** could selectively and specifically target lipid droplets. Hu *et al.* utilized the same mechanism to design a mitochondria-targeted fluorescent probe** 30** by conjugating dicyano-vinyl with benzopyran-chromone (Figure 7) 58.

Lysosomes are the circulating centers of cells that catalyzed the decomposition of a variety of waste such as proteins, nucleic acids, carbohydrates, lipids, and cell debris. Zhang *et al.* designed naphthalimide-hemicyanine-based fluorescent probe **31** for selective sensing of ONOO^-^ (Figure 7) 59. Morpholine group was introduced to** 31** as a lysosome-targeting unit. Owing to the strong oxidizing and nucleophilic ability of ONOO^-^, the C=C double bonds of **31** could be cleaved, and therefore, the fluorescence of **31** at 510 nm was enhanced. The experimental results revealed that **31** exhibited high selectivity over other ROS and RNS. Considering that the hemicyanine unit may respond to the HS^-^ or HSO_3_^-^, the authors further verified by selective experiments, and the results demonstrated that HS^-^ or HSO_3_^-^ did not interfere with the fluorescent intensity of **31**. It was noteworthy that the rotation of the double bond was limited with the solution viscosity increasing, and the fluorescence intensity of **31** also increased.

Because of the extensive applications of long-emission-wavelength probes in biological systems, a large number of related probes have been developed. Wang *et al.* developed a turn-off probe **32** based on benzothiazole-derived cyanine (Figure 7) 60. Li *et al.* developed a ratiometric NIR fluorescent probe **33** based on hemicyanine dye, with mitochondria-targeting abilities (Figure 7) 61. The C=C bond was cleaved through oxidization by ONOO^-^, and the π-conjugate effect of **33** was weakened at 742 nm, owing to the decrease in fluorescent intensity and enhanced at 487 nm. The fluorescence intensity ratio (*F*_487 nm_/*F*_742 nm_) was enhanced up to 436-fold. **33** could locate the mitochondria with a colocalization coefficient of 0.95. In addition, the authors elaborated on the influence of substituents on the emission wavelength of the probe. Furthermore, probe **33** could specifically target the mitochondria of living cells and was therefore used to assess the mitochondrial oxidative stress status in cells and mice. Also based on hemicyanine dye, James *et al.* synthesized fluorescent probe **34** (Figure 7) 62.

On the basis of the emission wavelength in the NIR region (610-800 nm), adding the ratiometric character can avoid the shortcoming of single-molecule probe to a greater extent. Lu* et al.* N-methyl benzothiazole and triphenylamine derivatives via vinyl to synthesize probe** 35** (Figure 7) 63. By destroying of the π coupling of the alkene moiety, **35** could quantitatively detect ONOO^-^ in the mitochondria with high selectivity. In the solution of water/glycerol (2/3, v/v), the fluorescence emission intensity ratio of *F*_485_/*F*_650_ was linearly related to the concentration of ONOO^-^. **35** could also respond to viscosity due to the presence of C=C double bonds. Thus, the authors concluded that probe was ratiometrically sensitive to mitochondrial viscosity changes and successfully applied the probe to detect the ONOO^-^ in the mitochondria of living cells and rat tissue.

Wang *et al.* applied the Knoevenagel condensation reaction between boron dipyrromethene (BODIPY) and two trans-cinnamaldehyde molecules to prepared probe **36** (Figure 7) 64. When 8.0-equal amount of ONOO^-^ was added to the solution of **36**, the ratio of fluorescence emission intensity (*F*_510_ /*F*_606_) increased by about 50-fold, and the color changed from red to green, which relied on ONOO^-^ induced diene oxidation. The LOD of ONOO^-^ was calculated to be 150.54 nM. The ratiometric and long-wavelength-emitting feature of **36** enabled it to be successfully applied in the study of ONOO^-^ in immune-stimulated macrophages and ONOO^-^ generation in phagosomes was then quantified via high-throughput flow cytometry analysis. You *et al.* employed diene as the recognition unit to devise and synthesize probe **37** (Figure 7) 65. **37** was based on the modified rhodamine as a fluorescent scaffold, and its emission wavelength extended to NIR region. Subsequently, by imaging of ONOO^-^ in acute inflammation mice model using probe **37**, the authors investigated the fluctuations in ONOO^-^ levels in a rheumatoid arthritis (RA) model of mice and assessed the response of the RA treatment with methotrexate.

By condensing fluorophore acedan and an indolium derivative, Lv *et al.* developed a coinstantaneous NIR- I window and ratiometric fluorescent probe **38** with both two-photon and mitochondria-targeting abilities (Figure 7) 66. In the range of 5-50 μM, the ratio of fluorescence intensities (*I*_535_/*I*_718_) of **38** displayed a good linear relationship with the concentration of ONOO^-^ due to the cleavage of the C=C double bond.** 38** exhibited high sensitivity to ONOO^-^ with an LOD of 85 nM. The authors further evaluated the imaging ability of **38**. They observed that the probe had two-photon imaging capability, could target cellular mitochondria, and could determine the degree of idiopathic pulmonary fibrosis progression by tissue imaging.

Using an HD dye and silicon-based rhodamine form a donor -acceptor pair and linking by short flexible piperazine-containing carbon chains, Tan *et al.* synthesized FRET-based ratiometric fluorescent probe **39**, with NIR and mitochondria-targeting ability (Figure 7) 67. The fluorescent intensity ratio (*F*_680 nm_/*F*_750 nm_) was linearly related to the concentration of ONOO^-^ (0-10 µM), with a detection limit of 0.36 µM. In addition, co-localization experiments proved that **39** possessed the ability to selectively detect ONOO^-^ in the mitochondria. Based on the above results, the authors finally used **39** to detect cisplatin (CIS) -induced changes in ONOO^-^ levels in the kidney.

Due to the strong oxidation and nucleophilic ability of ONOO^-^, the electron-rich conjugated C=C double bond in the probe was prone to oxidative cleavage, thereby realizing the detection of ONOO^-^. The C=N double bond exhibits similar properties as the C=C double bond and could be used as a response site for ONOO^-^ detection. Zhang* et al.* developed probe **40** based on the conjugation of 2-hydrazyl pyridine and 3-(benzo [D] thiazole-2-yl) -2-hydroxy-5-methylbenzaldehyde (Figure 7) 68. Before reacting with ONOO^-^, the fluorescence intensity of **40** was extremely weak because of the decay process of excited states by C=N isomerization. After reaction with ONOO^-^, the C=N bond was oxidized to an aldehyde, and a very strong fluorescence was observed at 524 nm. Furthermore, the probe had low cytotoxicity and high biocompatibility, which that enabled it to be used for the detection of intracellular ONOO^-^ in HeLa cells.

**Figure 8:**
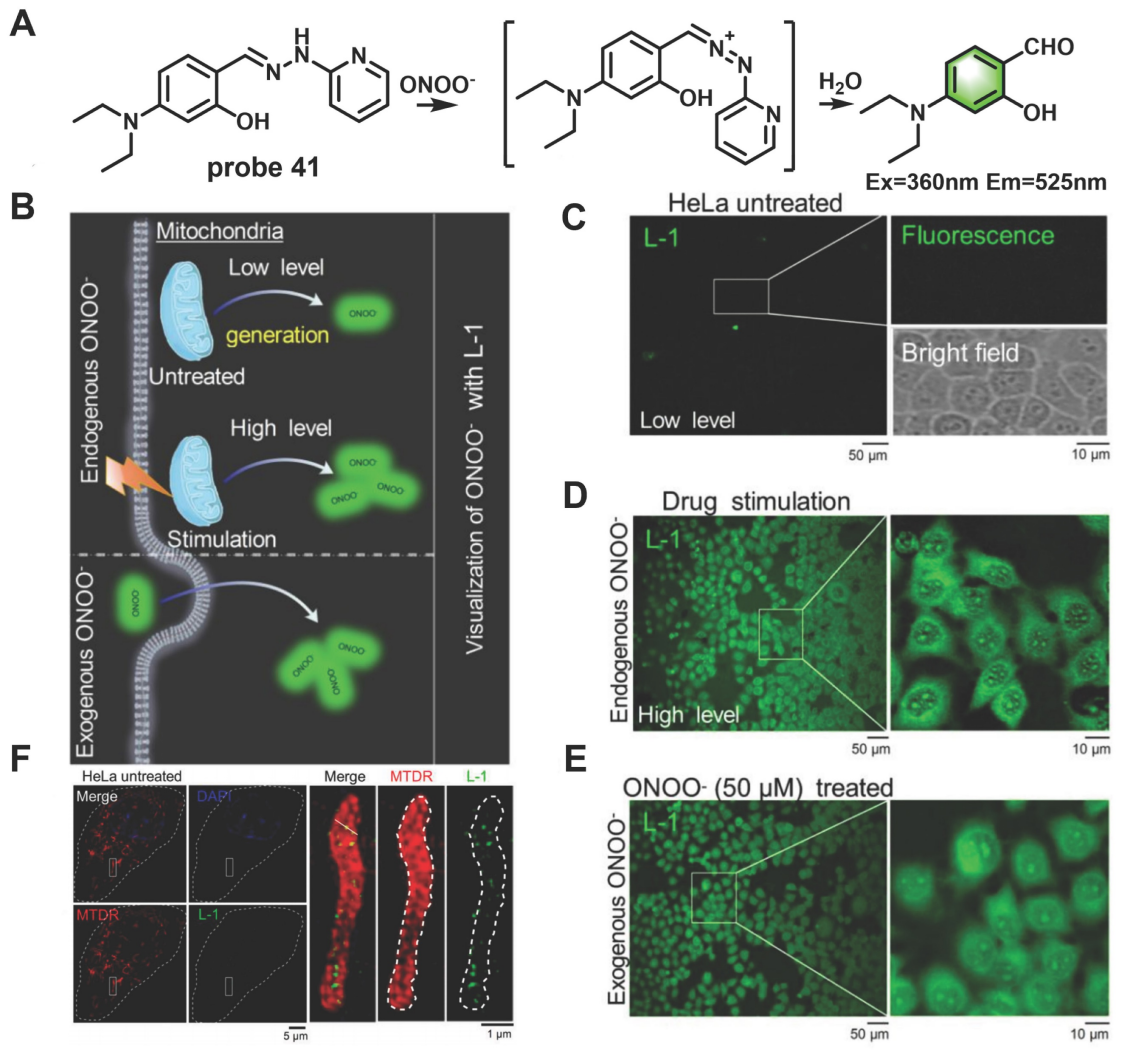
(A) The mechanism of probe **41** for sensing ONOO^-^. (B) Proposed ONOO^-^ visualization mechanisms for probe **41** in living cells. (C) Confocal fluorescence images of HeLa cells incubated with probe **41** (10 μM) for 30 min at 37 ℃, Zoom-in images of the regions of interest are presented in white rectangles; the upper right includes a fluorescent image, and the lower right presents a bright-field transmission image. (D) Fluorescence images of HeLa cells incubated with lipopolysaccharide (LPS, 1 µg/mL) and interferon-γ (IFN-γ, 100 ng/mL) for 10 h. (E) Fluorescence images of HeLa cells incubated with probe **41** (10 μM) for 30 min, and then with exogenous ONOO^-^ (50 μM) for 30 min. (F) Overlapping images of probe** 41**-stained ONOO^-^, Mito-tracker-deeper-red-stained mitochondria, DAPI-stained nucleus in untreated HeLa cells, zoom-in images of regions of interest in white rectangles representing the probe **41** and mitochondria. Adapted from 69 with permission.

In recent years, ultrahigh-resolution microscopy techniques such as structured illumination microscopy (SIM) have received extensive attention. This technique can help visualize the dynamic changes in sub-organelles at the nanoscale. Wang *et al.* skillfully designed a novel “mine-sweeping” probe **41** based on the condensation of an aldehyde group with 2-hydrazine pyridine (Figure 8) 69. **41** was “buried” in the cell matrix, and in the absence of ONOO^-^, it displayed almost no fluorescence signal due to C=N isomerization and the presence of PET from secondary amine groups to fluorophores. The C=N bond was cleaved after **41** reaction with ONOO^-^, and the fluorophore was released, resulting in a strong fluorescence being emitted at 525 nm. The use of **41** in SIM imaging of the exogenous and endogenous ONOO^-^ in HeLa cells was demonstrated (Figs. 8D, 8E). Furthermore, the method effectively solved the problem of non-specific staining of the probe and revealed for the first time that ONOO^-^ formation was bound to the crista of living cells by SIM imaging (Figure 8F).

### Probes based on hydrazides

**Figure 9:**
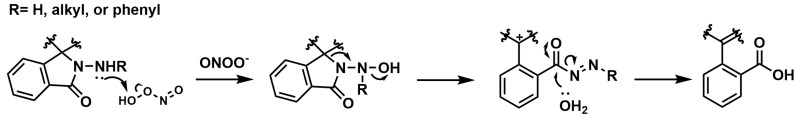
The design strategies based on hydrazides.

**Figure 10:**
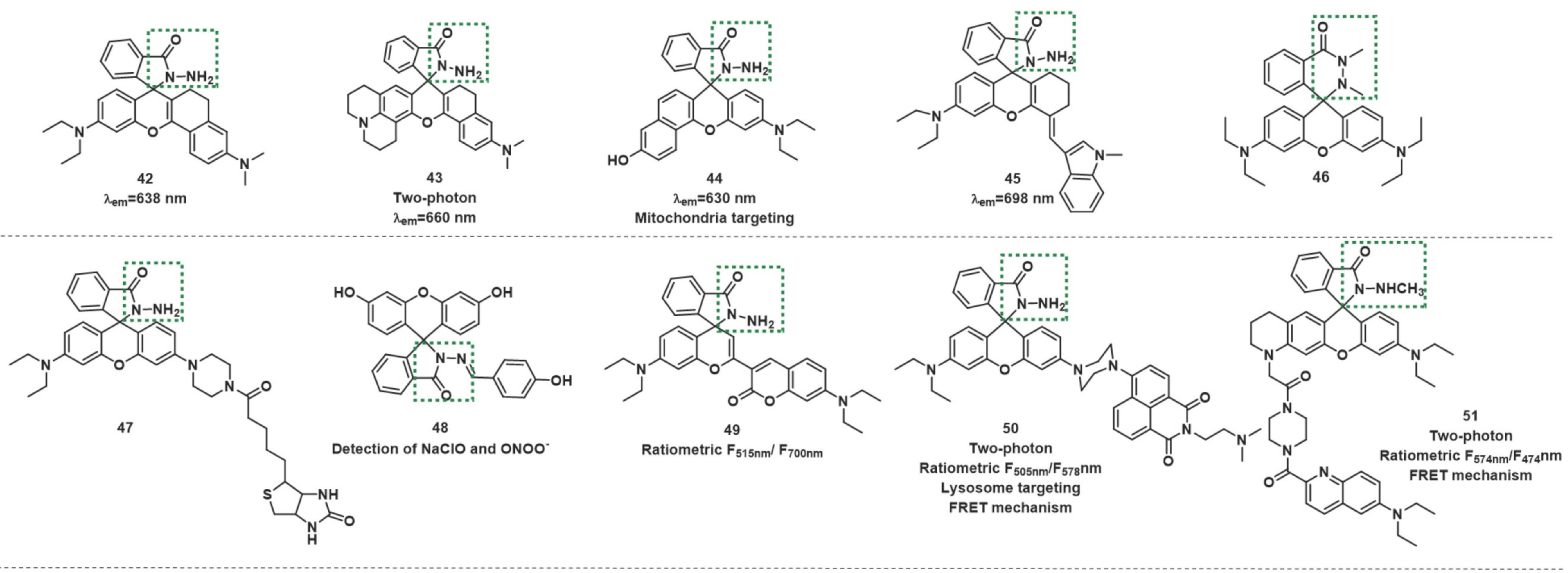
The structures of hydrazides-based fluorescent probes (**42**-**51**) for ONOO^-^ detection. The green boxes indicate the ONOO^-^ response unit.

In recent years, a growing number of rhodamine-type dyes with good stability and long absorption/emission wavelength have been successfully applied to fluorescence probes and imaging for detection. Incorporation of the hydrazide group into rhodamine derivatives was an ideal strategy to create probes that could selectively detect ONOO^-^ over other ROS/RNS. Probes designed based on this strategy have almost no fluorescence or only weak fluorescence, which attribute to the inherent structure of a spironolactone. After reaction with ONOO^-^, the non-conjugated form of spironolactone ring turned into the planar conjugated form, the functional group of hydrazide was converted to a carboxyl group, accompanied by a significant change about the fluorescence spectrum (Figure 9). Examples of this class of probes are depicted in Figure 10, and the spectroscopic properties of the end products are summarized in Table 3.

In 2017, Yoon *et al.* developed the far-red emissive probe **42** based on the above mechanism (Figure 10) 70. **42** was colorless and almost nonfluorescent. After reaction with ONOO^-^, which forced the spirolactam ring-opening, the fluorescence at 638 nm was discernibly enhanced. The authors noted a good linear relationship in the intensity of fluorescence at 638 nm and the concentration of ONOO^-^ in the range of 0-34 µM, and the LOD of the probe was 45 nM. The probe was successfully applied to imaging the exogenous and endogenous ONOO^-^ in living RAW264.7 cells and HeLa cells. More importantly, probe **42** was also used to detect endogenous ONOO^-^ generated in *Pseudomonas aeruginosa*-infected mouse bone marrowderived neutrophils. Likewise, probes **43**-**48** have been used for the same (Figure 10) 71-76.

The next year, Feng *et al.* designed dual-channel NIR fluorescent probe **49** (Figure 10) 77. **49** contains a semirhodamine structure and a coumarin fluorophore. The semirhodamine part used the same spirocyclization mechanism as a rhodamine dye. When **49** was in the spiro-opening form, it emitted fluorescence in the NIR region. However, because of the coumarin structure in the molecule, **49** still exhibited a strong visible fluorescence when the semirhodamine structure was in the spirocyclic form at 515 nm. Upon addition of ONOO^-^ into **49** solutions, the fluorescence intensity markedly decreased at 515 nm (λ_ex_=430 nm) and significantly increased at 700 nm (λ_ex_=640 nm) significantly increased with the emission color changing from green to red. **49** was found to have excellent selectivity and sensitivity to ONOO^-^ and demonstrated promising potential for *in vitro* and *in vivo* detection of ONOO^-^.

Two-photon ratiometric fluorescent probes **50** and **51**, based on the FRET mechanism were synthesized by Zhou *et al.* and James *et al.* respectively (Figure 10) 78, 79. Which all selectively reacted with ONOO^-^ against other ROS including ClO^-^. **50** and **51** chose naphthalimide and quinolone derivative as the two-photon fluorophore respectively. In addition, probe **50** introduce a dimethylamino as lysosome-targeting group, which could stain lysosomes specifically in living cells for ratiometric imaging. In these two probes, rhodamine derivatives act as energy acceptors. For a spiral ring structure, the absorption spectrum of acceptors was weak, which overlapped poorly with the emission spectrum of donors. Therefore, FRET was prohibited, and only the emission spectrum of donors was displayed. After reaction with ONOO^-^, the fluorescence of rhodamine was released due to the recovery of spirocyclization opened-form. Both of the probes were found to have the capability of deep tissue penetration imaging and high-resolution ratio imaging.

### Probes based on N-dearylation

**Figure 11:**
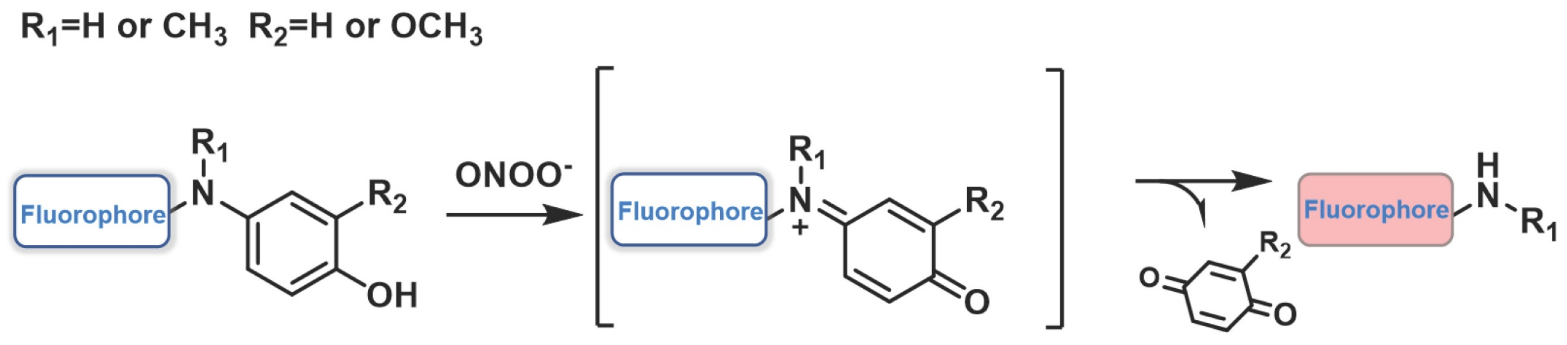
The design strategies based on N-dearylation.

**Figure 12:**
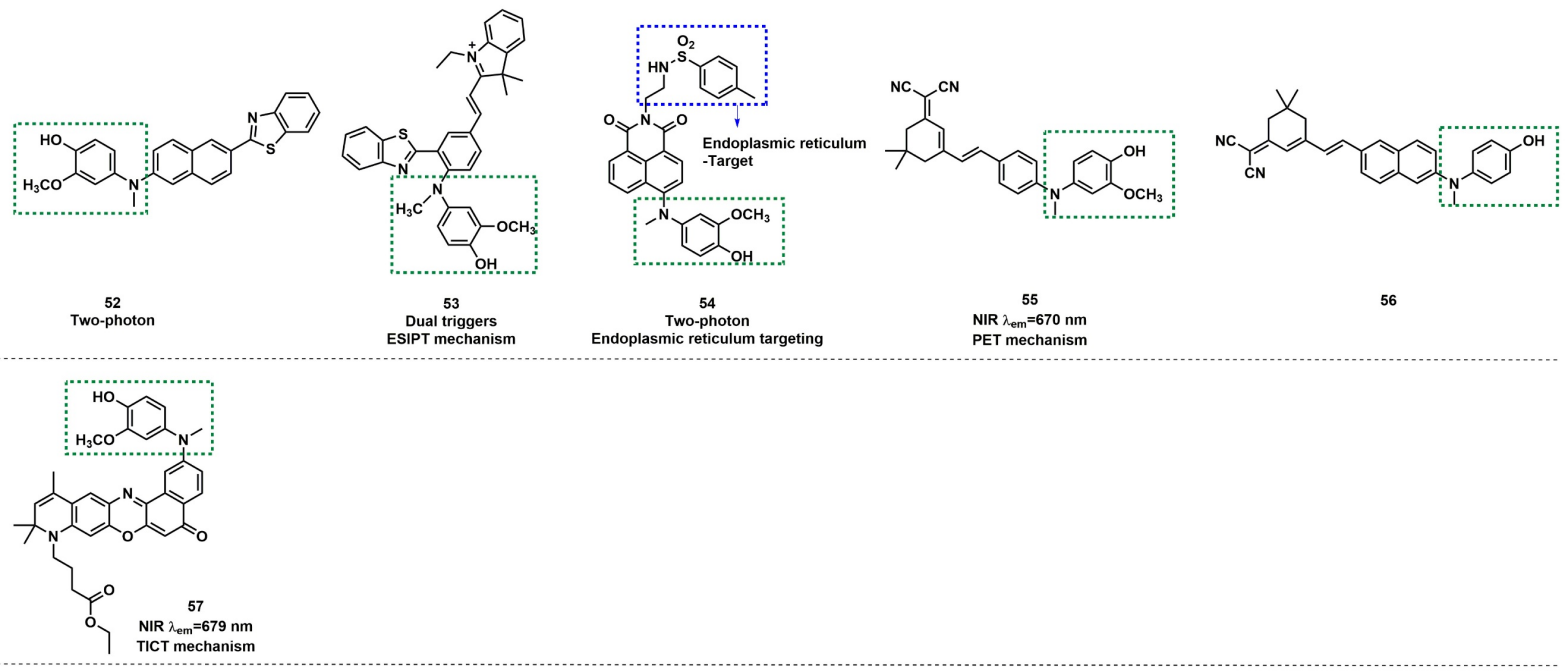
The structures of oxidative N-dearylation fluorescent probes (**52**-**57**) for ONOO^-^ detection. The green boxes indicate the ONOO^-^ response unit, and the blue boxes indicate the targeting moiety.

The common ONOO^-^ response sites, various substituted p-aminophenols, such as 3-hydrogen/methoxy/methyl and 3, 5-dimethyl, were integrated into the fluorophore to form fluorescent probes with specific ONOO^-^ responses. Due to the highly electron-rich nature of the p-aminophenol group, the fluorescence of the fluorophore could be effectively quenched by the PET mechanism, as illustrated in Figure 11. When reacted with ONOO^-^, strong fluorescence would be restored on account of cleavage of the p-aminophenols or *p*-aminophenol derivatives in the probes. Based on these, some excellent ONOO^-^ probes have been designed and synthesized. The examples of this class of probes are presented in Figure 12, and the spectroscopic properties of the oxidation products are summarized in Table 4.

Based on naphthozothiazole skeleton as fluorophore, Yoon *et al.* reported a two-photon probe **52** with good sensitivity and selectivity for ONOO^-^ (Figure 12) 80. **52** itself exhibited very weak fluorescence, but after reaction with ONOO^-^, the fluorescence intensity at 500 nm was enhanced. **52** demonstrated excellent specificity and LOD as low as 35 nM. In addition, the probe was found to possess excellent two-photon properties and could be used to image ONOO^-^ in rat hippocampus slices, to a depth of up to 120 µm. Using phenylbenzothiazole as a skeleton, Li *et al.* synthesized a novel fluorescent probe **53** (Figure 12) 81. The selectivity and specificity of **53** to ONOO^-^ were notably enhanced by introducing N-phenyl group and merocyanine group simultaneously on the fluorescent skeleton as two response sites. When the concentration of ONOO^-^ was small, the *p*-aminophenol would transform to a simple N-H group to restore the fluorescence. With the increase in ONOO^-^ concentration, the C=C double bond was oxidized and broken into carboxyl groups. Furthermore, the red emission was attenuated, whereas the green emission was enhanced.

Li *et al.* employed 1,8-nephthalimide as the fluorophore and integrated 4-amino-2-methoxyphenol to serve as the ONOO^-^ receptor *p*-toluenesulfonamide as the ER targeting group to synthesize probe** 54** (Figure 12) 82. Prior to contact with ONOO^-^, **54** displayed weak fluorescence owing to the PET effect from the phenol group to the naphthalimide moiety. However, when different concentrations of ONOO^-^ were added, the fluorescence intensity of **54** at 540 nm gradually increased. **54** was found to have high selectivity and sensitivity to ONOO^-^ and an LOD as low as 8.3 nm. Furthermore, it had excellent endoplasmic reticulum (ER) targeting and revealed the changes in ONOO^-^ concentration levels in the ER of living cells and *Caenorhabditis elegans* by two-photon imaging. Most importantly, the probe was successfully applied for imaging the ONOO^-^ in Parkinson's disease models, indicating its potential application for revealing the fundamental roles of ONOO^-^ in ER stress-related diseases.

**Figure 13:**
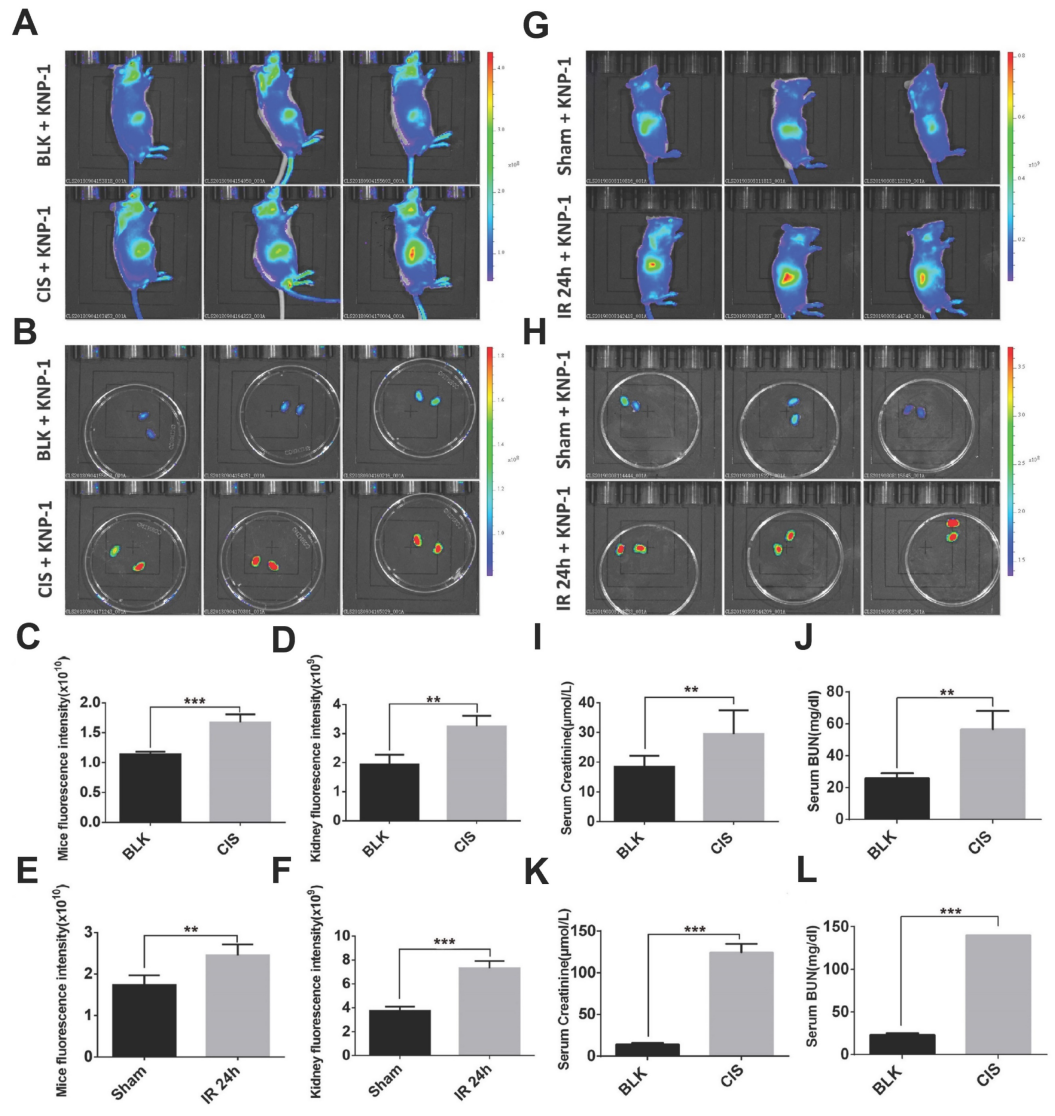
Imaging ONOO^-^ formation in CIS- or IR-induced AKI in live mice. Representative images of mouse (A) body and (B) kidney 48 h after treatment with CIS or saline, followed by KNP-1 (Probe **57** 0.5 mg/kg) intravenously. Representative images of mouse (G) body and (H) kidney suffering from bilateral renal IR and sham operation (Sham), followed by Probe **57** (0.5 mg/kg) intravenously. The graphs of the fluorescence statistics of (A), (B), (G), and (H) are shown in (C), (D), (E), and (F). The statistical graphs of sCr/BUN of (A, B) and (G, H) are depicted in (I, J) and (K, L). Adapted from 85 with permission.

Based on the same response site and reaction mechanism, the research group also synthesized NIR probe **55** using a dicyanoisophorone as a fluorophore (Figure 12) 83. With a similar type of fluorophore structure as **55**, Li *et al.* designed probe** 56** (Figure 12) 84. Li *et al.* selected Nile red derivative, an inherently kidney-targeting NIR fluorophore to synthesize probe **57** (Figure 12) 85. They found that the probe exhibited desirable kidney distribution after intravenous administration and was fluorescent only after activation by ONOO^-^. Owing to these properties, the probe yielded excellent kidney contrast imaging results. As depicted in Figures. 13A and 13B, the mouse body and kidneys with AKI showed higher probe fluorescence than those without AKI. As evident in Figs.13g and 13h, compared with the sham group, probe fluorescence was significantly higher in ischemia-reperfusion (IR) 24 h mouse body and kidneys. The above evidence confirmed that probe **57** was capable of detecting both nephrotoxin-induced and IR injury- induced AKI in live mice.

### Probes based on ketones

**Figure 14:**
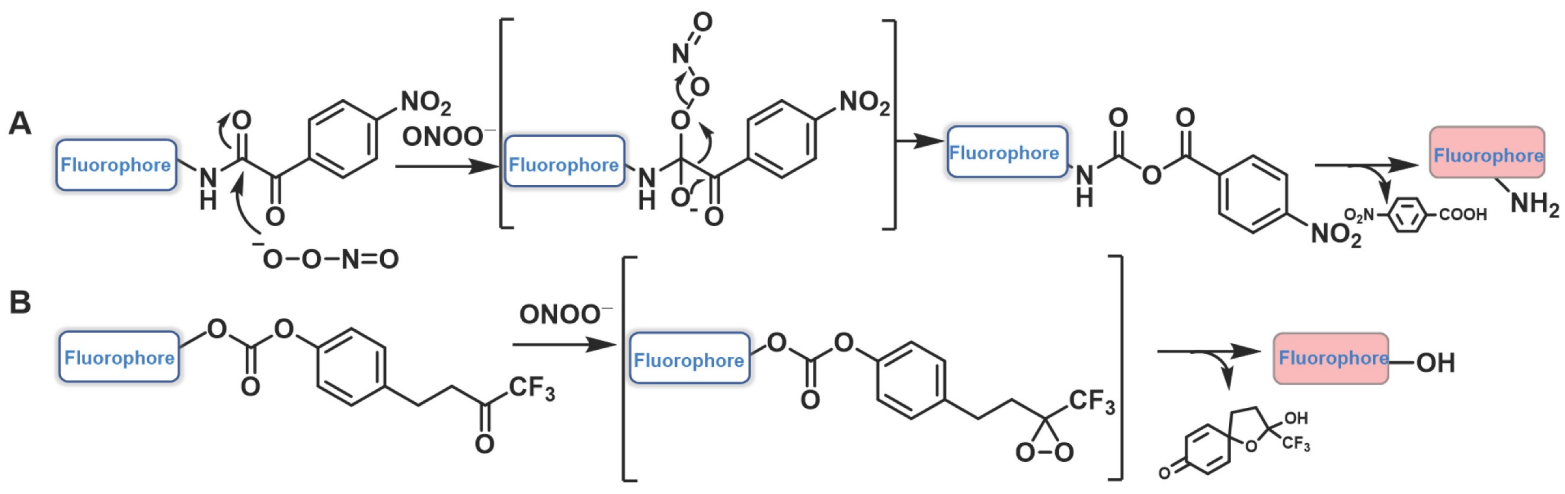
The design strategies based on α-ketoamide.

**Figure 15:**
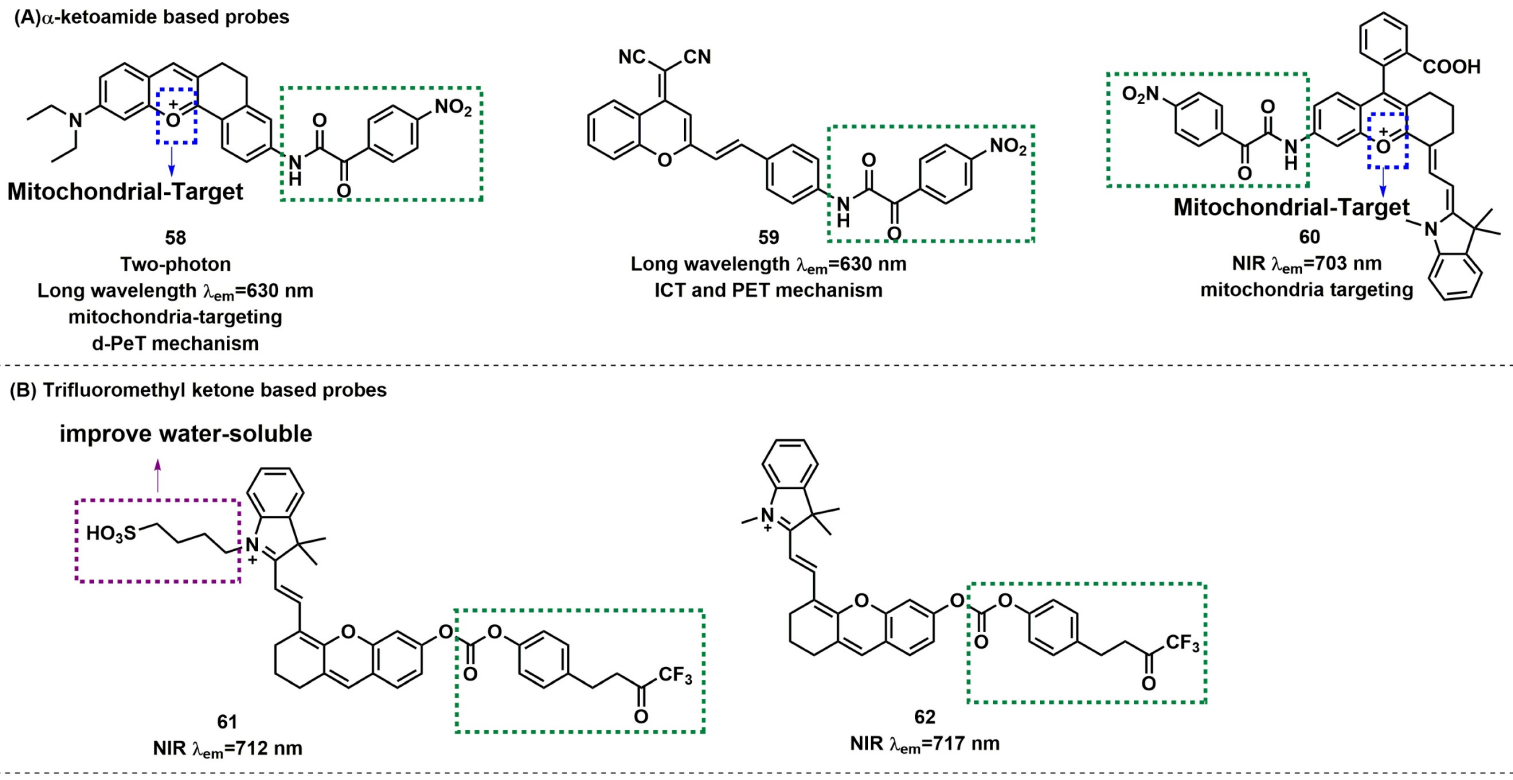
The structures of ketones-based fluorescent probes (**58**-**62**) for ONOO^-^ detection. (A) α-ketoamide-based fluorescent probes** 58**-**60**. (B) Trifluoromethyl ketone-based probes** 61**-**62**. The green boxes indicate the ONOO^-^ response unit, and the blue boxes indicate the targeting moiety.

α-ketoamide was introduced as a recognition moiety to the probes for ONOO^-^-specific recognition. These probes displayed very weak fluorescence or no fluorescence because the electron-withdrawing properties of α-ketoamide moiety attenuated the electronic transfer effects such as intramolecular charge transfer (ICT) and photoinduced electron transfer (PET). When ONOO^-^ was present, the bridge of α-ketoamide was cleaved and released the fluorophore, thereby restoring fluorescence (Figure 14a). Trifluoromethyl ketone group, with a similar structure to α-ketoamide, could also be used as the specific response group of ONOO^-^ (Figure 14b). This class of probes is illustrated in Figure 15, and the spectroscopic properties of the detectable products are summarized in Table 5.

Due to the large rigid conjugated system and lipophilic cation moiety, Tang *et al.* selected** TPNIR-NH_2_** as the optical fluorophore to devise two-photon imaging and NIR-emissive fluorescent probe **58**, which also had mitochondria-targeting characteristic (Figure 15) 86. **58** exhibited good linearity in a plot of the intensity of fluorescence at 630 nm and the concentration of ONOO^-^ (0-5 equiv.). By using probe **58**, the authors confirmed the ability to track the subtle concentration variations of mitochondrial ONOO^-^ in cardiomyocytes and cardiac tissues. Subsequently, the authors also demonstrated that ONOO^-^ burst emerged in the early stage after anthracycline administration and played a crucial role in initiating and promoting cardiomyocyte apoptosis. Using **DCM-NH_2_** as a fluorophore, Liu *et al.* designed far-red fluorescent probe** 59** (Figure 13) 87. After reaction with ONOO^-^, **59** had a fluorescence maximum at 630 nm accompanied with color changes from light yellow to orange, which was detectable with the naked eye. **59** was successfully used to detect changes in endogenous ONOO^-^ levels in macrophage J774A.1 cells.

To resolve the issue of false signal in the practical application of ONOO^-^ fluorescent probes and increase their sensitivity, Yuan *et al.* purchased or synthesized **60** kinds of NIR dyes and selected HD-NH_2_ as the fluorophore with the most excellent properties among them. Probe **60** was synthesized by introducing a benzoic acid group and ketoammonia into HD-NH_2_ (Figure 15) 88. Benzoic acid group could improve the selectivity by preventing attack by other species. In PBS/EtOH (v/v, 8/2, pH 7.4) buffer solution, addition of ONOO^-^ (0-15 µM) to this probe led to a significant NIR fluorescent enhancement (seven-fold) and a red-shifted absorption from 611/661 to 627/678 nm. **60** was successfully used to monitor the effectiveness of remediation medicines for APAP-induced hepatotoxicity and to assess the liver protective effects of hepatoprotective medicine.

With trifluoromethyl ketone as the response site, a cascade oxidation-elimination reaction occurred after contacting with ONOO^-^, which released the phenolic hydroxyl group in the fluorophore and enhanced the fluorescence. Pu *et al.* reported a new NIR fluorescent probe **61**, composed of a water-soluble hemicyanine dye caged with a trifluoromethyl ketone moiety (Figure 15) 89. The zwitterionic structure of the hemicyanine component in **61** ensured its good water solubility. In addition, the fluorescence at 712 nm was significantly enhanced by the addition of ONOO^-^ and was ~59 times compared with the initial state. A good linear correlation existed between the fluorescent intensity and ONOO^-^ concentration in the range of 0-8 µM with an LOD of 53 nM. Notably, probe **61** was systemically injected into living mice for NIR fluorescence and photoacoustic (PA) dual-modal imaging of ONOO^-^. The same research group produced NIR fluorescent probe **62** for ONOO^-^ sensing by utilizing a similar strategy (Figure 15) 90.

### Probes based on chalcogenides

**Figure 16:**
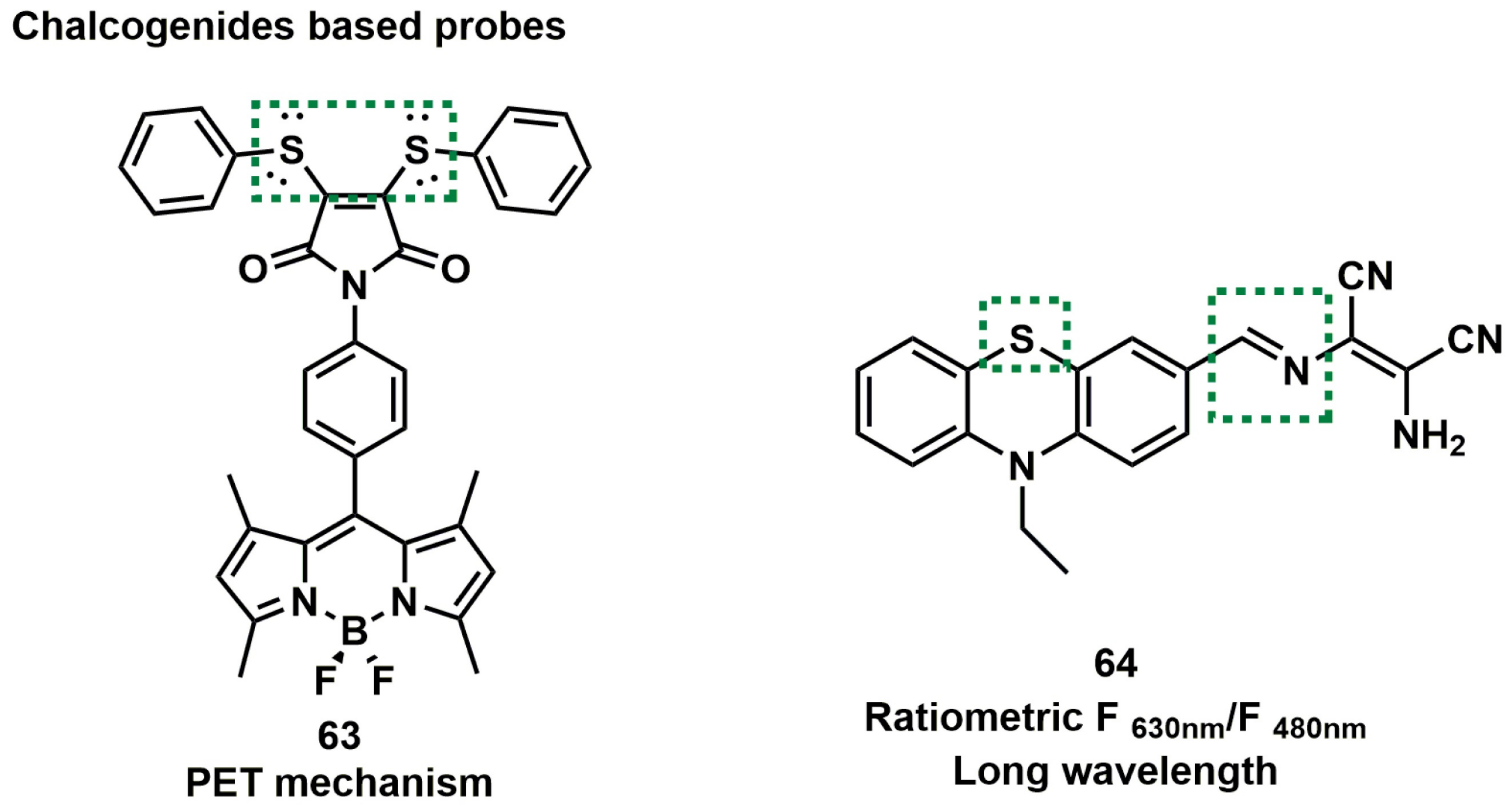
Chalcogenides based probes **63**-**64**. The green boxes indicate the ONOO^-^ response unit.

The electron- rich characteristics of thioether enables it to react with ONOO^-^ rapidly, markedly changing the electron cloud density of sulfur atom. Therefore, upon the introduction of thioether groups into the probe, after the reaction with ONOO^-^, the fluorescence intensity would lead to obviously changes. That was also one of the design strategies in the traditional ONOO^-^ detection fluorescent probe. Their structures and properties are summarized in Figure 16 and Table 6.

[3, 4-*bis* (phenylthiol)] was incorporated into maleimide-4-phenyl moiety which was, in turn, located in the *meso*-position of the BODIPY system. Based on this, Churchill *et al.* developed probe **63** (Figure 16) 91, which exhibited neligible fluorescence, owing to the occurrence of the PET mechanism. Addition of ONOO^-^ into **63** solution, resulted in an 18-fold fluorescence emission enhancement because the thioethers were oxidized to sulfones to block the PET process. High concentrations of O^2-^ were indeed a potential interferent for ONOO^-^. In addition, owing to the thiophilic nature of Hg^2+^, the presence of Hg^2+^ in the detection of ONOO^-^ would interfere with the detection results.

Because phenothiazine has the reducibility of the sulfur atom, Shan *et al.* chose it as the fluorophore backbone and synthesized probe **64**. They introduced a C=N double as another specificity response site to ONOO^-^ at the same time (Figure 16) 92. After reaction with different amounts of ONOO^-^, the changed of the changes in the two emission bands were not synchronized, which might be attributed to the multi-step oxidation of the probe by ONOO^-^. The probe could be used to monitor the up-regulation of ONOO^-^ in cancer cells and normal cells during 5-fluorouracil treatment.

### Other methods

**Figure 17:**
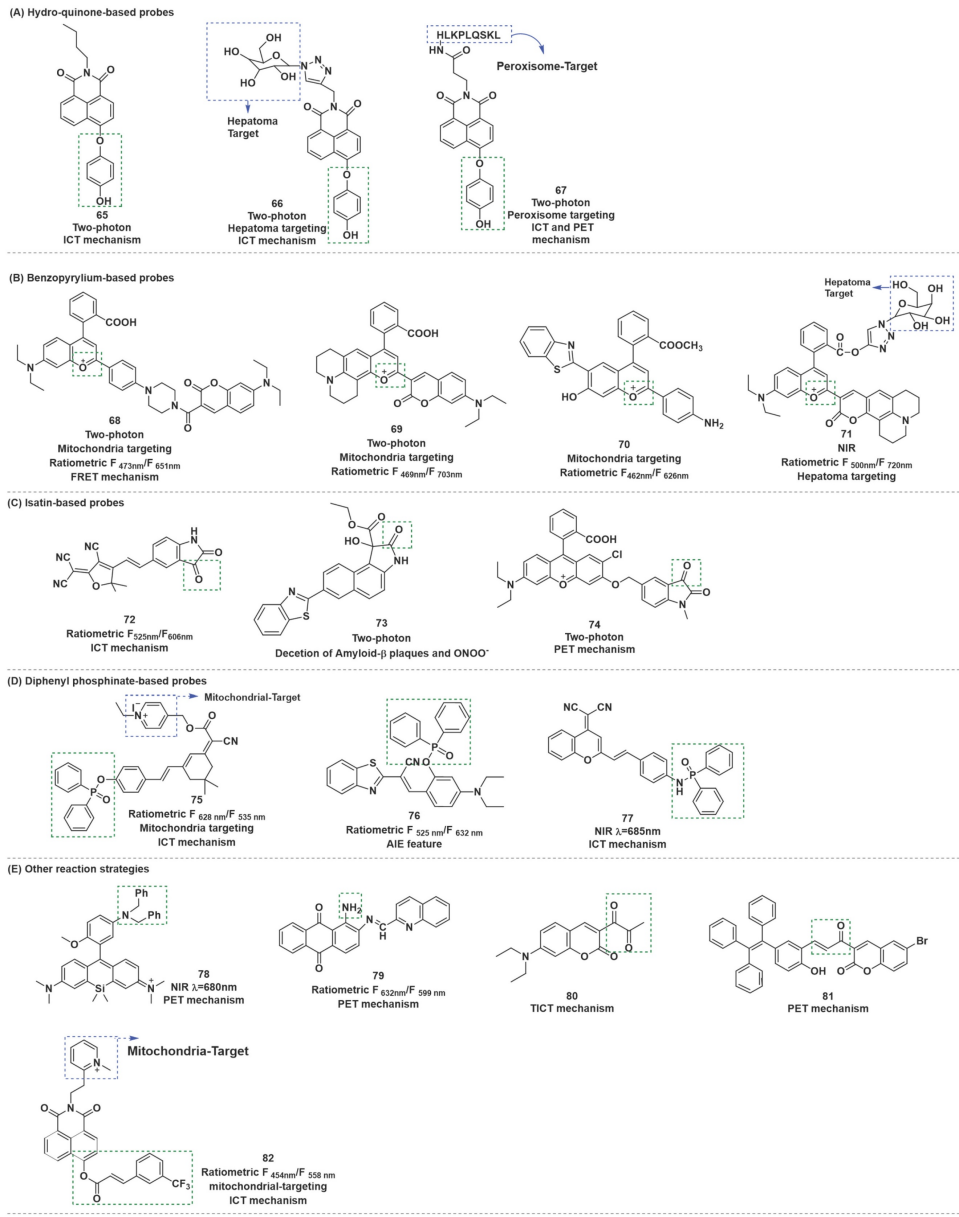
The structures of fluorescent probes based on other methods (**65**-**83**) for ONOO^-^ detection. (A) Hydro-quinone-based fluorescent probes **65**-**67**. (B) Benzopyrylium-based probes** 68**-**71**. (C) Isatin-based probes **72**-**74**. (D) Diphenyl phosphinate-based probes **75**-**77**. (E) Probes based on other reaction strategies **78**-**82**. The green boxes indicate the ONOO^-^ response unit, and the blue boxes indicate the targeting moiety.

As depicted in Figure 17, in addition to the methods listed above, several fluorescent probes have been developed for ONOO^-^ sensing based on other mechanisms. A comparison of the spectroscopic properties, sensitivity and applications of representative ONOO^-^ probes is presented in Table 7.

Hydroquinone was employed as a recognition receptor due to its excellent specificity to ONOO^-^. Different research groups have designed and synthesized probes with this recognition receptor. After the probes reacted with ONOO^-^, hydroquinone moiety was removed by oxidation, and the ICT effect from hydroxyl group to fluorophore occurred. Therefore, the fluorescence recovery of fluorophore, and could realize the detection of ONOO^-^. At the same time, HClO and H_2_O_2_ did not interfere with the detection results. Based on this mechanism, Zhu *et al.* synthesized probe **65** (Figure 17) 93. To optimize the structure of **65**, the same research group synthesized probe **66** by introducing galactose as a hepatoma-specific targeting group and a hydrophilic group (Figure 17) 94. **66** exhibited optical properties similar to **65** and was successfully utilized for hepatoma-selective imaging. Based on the same fluorophores, response sites, and detection mechanism, Tang* et al.* prepared another two-photon fluorescent probe **67** (Figure 17) 95. Notably, utilizing probe **67**, the authors proved that ONOO^-^ was significantly up-regulated in the livers of mice during ALI.

**Figure 18:**
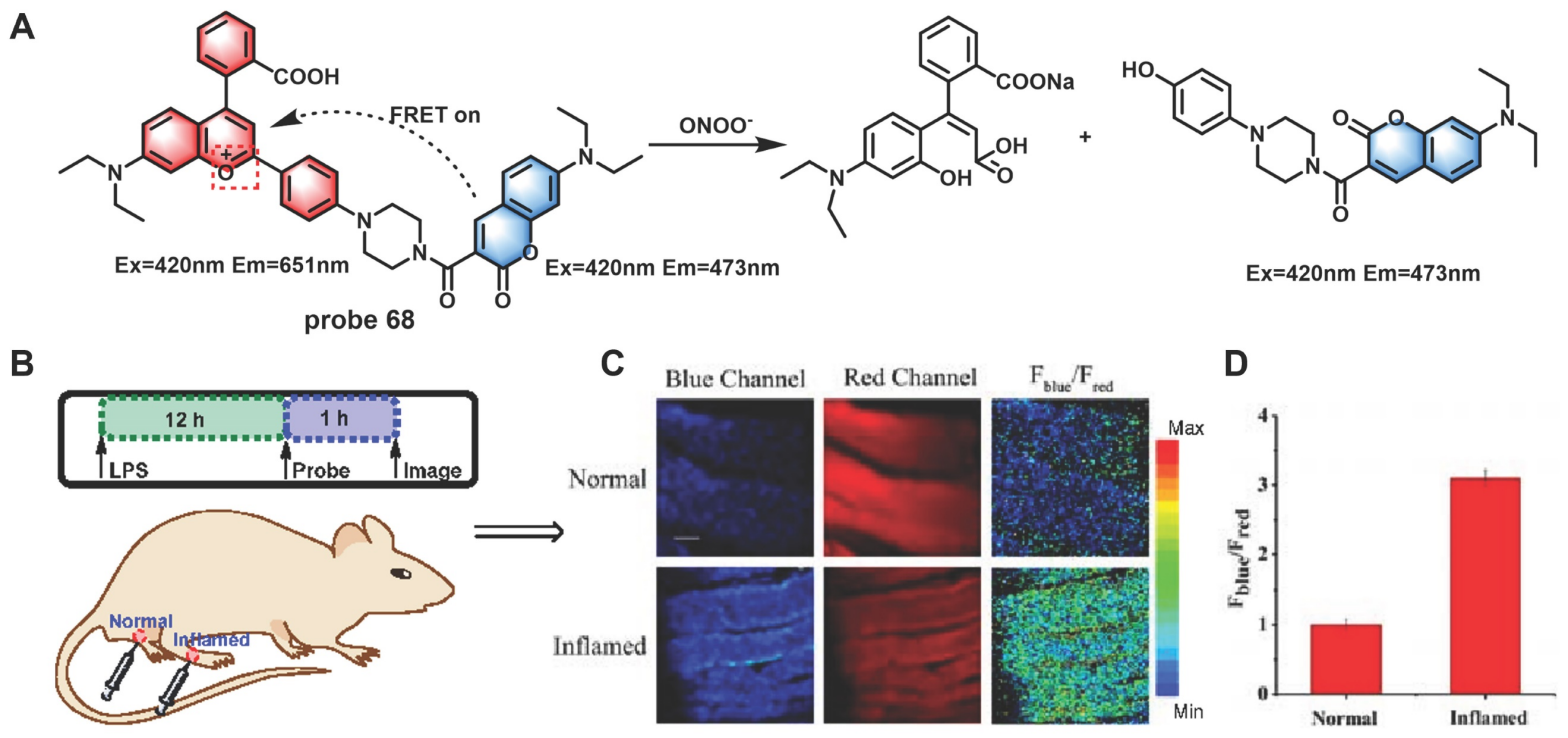
(A) Reaction between probe **68** and ONOO^-^. Two-photon confocal microscopic fluorescence images for detecting LPS-dependent ONOO^-^ generation in inflammation tissues via probe **68**. (B) 200 μL of LPS (1 mg/mL) was subcutaneously injected into the right leg of mice to cause inflammation. After 12 h, 20 μL of 500 μM probe **68** was subcutaneously injected in situ. After 1 h, the leg skin of mice was sectioned after being anaesthetized. (C) Fluorescence images of probe **68** in the normal and inflamed tissues. (D) Average *F*_blue_/*F*_red_ intensity ratios in panel B. Blue channel, λ_em_ = 460-500 nm; red channel, λ_em_= 605-680 nm. λ_ex_ = 800 nm. Scale bar: 200 μm. Adapted from 96 with permission.

Generally, destroyed-type probes are one of the chief modes for ratiometric ONOO^-^ detection. The carbonyl group of the benzopyrylium fluorophore could react with ONOO^-^, which could result in the breakage of the unsaturated bond and finally produce an olefine acid product to realize the purpose of detecting ONOO^-^ using a ratiometric probe. Based on this mechanism by combining dye-screening approach, Yuan *et al.* devised ratiometric fluorescent probes **68**, **69** and **70** for ONOO^-^ detection (Figure 17) 96-98. Benzopyrylium derivative and coumarin were combined in different forms to synthesize probes **68** and **69**. The results of cell imaging experiments revealed that **68** and **69** could be used to detect endogenous ONOO^-^ production in cells by either by one or two-photon fluorescent confocal microscopy. Moreover, probe **68** was found to be capable of monitoring ONOO^-^ produced by LPS stimulation in the inflamed mouse model. After being injected with LPS to induce inflammation, probe **68** was injected. After that, the mice were anesthetized, and the leg skin was sectioned for the latter two-photon fluorescence imaging. As shown in Figure 18, discernibly enhanced fluorescence of blue channel (λ_em_ = 460-500 nm) and a relatively weak fluorescence signal of red channel (λ_em_ = 605-680 nm) were observed, upon excitation at 800 nm in the inflamed tissue. **70** was a combination of long-wavelength fluorophore NH_2_-benzopyrylium dye and a short-wavelength fluorescence dye benzothiazole. It was developed to monitor ONOO^-^ *in vivo* by introducing the ester structure to enhance its membrane penetrability. In cell imaging experiments, **70** displayed outstanding selectivity and biocompatibility and enabled noninvasive visualization of ONOO^-^ generation in a different drug-induced ALI model.

Based on the same response mechanism, Li *et al.* designed hepatocyte-targeting ratiometric NIR fluorescent probe **71** for ONOO^-^ sensing*.* (Figure 17) 99. **71** was developed by grafting galactose onto a coumarin-benzopyrylium-based fluorophore. The galactose group in** 71** made the probe exhibit excellent hepatocyte-targeting ability. All four probes could target mitochondria in cells owing to their electropositive cation.

Isatin and its derivatives were found to be vulnerable to attack by ONOO^-^ after being linked to the electron-withdrawing fluorophores. ONOO^-^ nucleophilicity attacked the carbonyl of indoline-2, 3-dione, followed by intramolecular cyclization and rearrangement to afford 2-aminobenzoic acid derivatives, and then fluorophores were released through self-immolative 1, 6-elimination. By conjugating an isatin moiety with an electron-withdrawing tricyanofuran (TCF) moiety, Tian et al. designed probe **72** (Figure 17) 100. Probe **72** reacts with ONOO^-^ through an oxidative decarbonylation reaction to initiate light emission that can be observed instantly with high sensitivity and selectivity. More specifically, addition of ONOO^-^ to the solution of 72 generated a 36-fold increase in the emission peak at around 606 nm. **72** was highly selective and sensitive for ONOO^-^ compared with other active sulfur, oxygen and nitrogen.

Tang* et al.* developed two-photon fluorescent probe** 73** based on the benzothiazole-naphthalene derivatives (Figure 17) 101. In this probe, the oxindole functionality could specifically react with ONOO^-^, thereby achieving a highly sensitive and selective detection of ONOO^-^. Meanwhile, **73** could achieve an independent spectral response to Aβ plaques. Two key events, namely, ONOO^-^ stress and Aβ aggregation, were found to amplify each other through a positive feedback mechanism and jointly promote the occurrence and development of Alzheimer's disease (AD). This observation enabled the identification and visualization of these two pathological factors in AD through two independent fluorescence channels.

Liu* et al.* developed another two-photon probe** 74**, which also contained indoline-2, 3-dione moiety as the recognition domain (Figure 17) 102. Probe **74** was used to track the endogenously produced ONOO^-^ in living cells during the stress response to external stimulants. Furthermore, it could monitor the ONOO^-^ production in LPS-induced kidney injury of zebrafish. Benefiting from the two-photon excitable property of probe **74**, a high-fidelity 3D reconstructed image of local mouse cerebral microvessels was presented by TP-CLSM. In particular, the probe was applied for in vivo visualization of the profile of ONOO^-^ in microvessels of mouse brains with ischemic and hemorrhagic strokes.

Diphenylphosphinate or diphenylphosphinamide as the recognition moiety, was incorporated with fluorophore to form turn-on-type fluorescent probes. The ester bond or amide bond was broken after the probe reacted with ONOO^-^, and then the hydroxy or amino group was released and emitted strong fluorescence, owing to the restoration of the ICT progress.

Li *et al.* employed an isophorone derivative as the fluorescent group and pyridinium cation as the mitochondria targetable group to construct a ratiometric and long-wavelength fluorescent probe **75** by using the above strategy (Figure 17) 103. The contact of **75** with ONOO^-^ resulted in the disappearance of the initial emission peak at 535 nm, while a new far-red emission appeared at 628 nm. The ratio of *F*_6 nm_ was linearly dependent on the ONOO^-^ concentration in the range of 0 to 10 μM, and the LOD was 13.3 nM. **75** was successfully applied for ratiometric fluorescent imaging of in mitochondria of living cells as well as visualization of ONOO^-^ in zebrafish. Based on the same mechanism, with benzothiazolyl derivative as the fluorophore and well meanwhile as the AIE-active luminogen, Tang *et al*. designed probe **76**, composed of diphenylphosphinate as the recognition site (Figure 17) 104. Furthermore, the authors applied this probe successfully to ONOO^-^ imaging in live mice. With dicyanomethylene-benzopyran as a NIR TP fluorophore, diphenylphosphinamide group as a specific reaction moiety, Yu* et al.* synthesized probe **77** (Figure 17) 105. **77** was successfully applied to detect endogenous ONOO^-^ in live cells and in rat epileptic brain. The imaging results revealed that the increase in the ONOO^-^ level was closely related to severe neuronal damage in the brain under epilepsy and KA stimulation.

**Figure 19:**
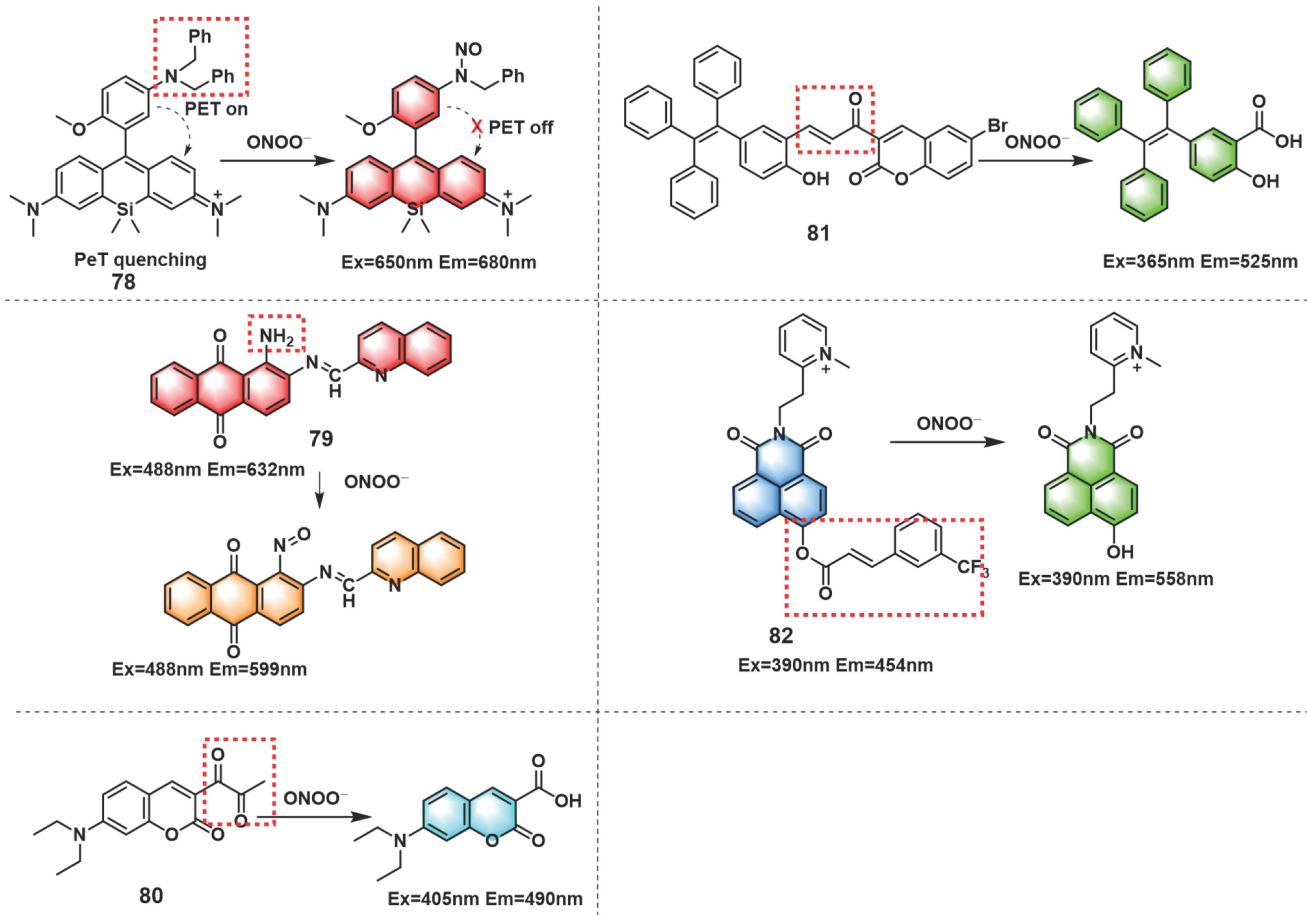
Other novel strategies mechanism. The red boxes indicate the ONOO^-^ response moiety.

Besides the reaction strategies described above, certain other novel reaction strategies have also been considered by scientists. For instance, amido could be oxidized to generate nitroso by ONOO^-^. Based on this, fluorescent probes with amino as recognition sites may have excellent selectivity and sensitivity to ONOO^-^. Wei *et al.* introduced amido into Si-rhodamine to develop NIR fluorescent probe **78** (Figure 19) 106. In the presence of ONOO^-^, **78** displayed an emission maximum at 680 nm when excited at 650 nm, which was successfully applied to exogenous and endogenous ONOO^-^ imaging in cells. The authors further used** 78** to evaluate the therapeutic effect of phenolic acid antioxidants on IR injury in EA.HY926 endothelial cells and the pathogenesis of diabetic nephropathy in activated pancreatic β-cells and diabetic rats. Dong *et al.* synthesized probe **79** with an extremely low background fluorescence using the same mechanism (Figure 19) 107.

The diketone group or α, β-unsaturated ketene of the synthesized fluorescent probes could be converted into their monoketone or carboxylic acid derivatives when contacted with ONOO^-^, resulting in fluorescence recovery of the probe. According to this fluorescence turn-on strategy, probes that respond to ONOO^-^ could be designed. Li* et al.* described a turn-on fluorescent probe** 80** for selective recognition of ONOO^-^ (Figure 19) 108. Finally, the probe was employed in the imaging of ONOO^-^ induced by drug or heat shock in RAW 264.7 cells.

Using α, β-unsaturated ketene as a recognition site for ONOO^-^, by linking a bromo-coumarin derivative to a tetraphenyl ethylene (TPE)-based motif, Tang *et al.* synthesized a turn-on fluorescent probe **81** (Figure 19) 109. Upon reaction with ONOO^-^, unsaturated ketene was rapidly oxidized and broken, and released TPE-COOH which presented aggregation-induced fluorescence. This chemical change resulted in a large enhancement of the fluorescence intensity at 525 nm. **81** evaluated and verified the protective effect of estrogen on myocardial cells during oxygen-glucose deprivation/reperfusion. Based on 3-(trifluoromethyl) cinnamic acid (3-TCA) as ONOO^-^ reaction site, Zhang *et al.* developed a highly selective and sensitive mitochondrial-targeted probe **82** (Figure 19) 110. Although **82** also contained unsaturated ketene structures similar to those in **81**, the response mechanisms of them were completely different. The ester bond between 3-TCA to the hydroxyl group of 1,8-naphthalimide was cleaved after **82** reacted with ONOO^-^, accompanied by a large red-shift of fluorescence emission from 454 nm to 558 nm and manifested obvious ratiometric fluorescence changes (18-fold). **82** could be utilized to detect endogenous ONOO^-^ fluctuations in the mitochondria.

## Nanoparticle (NP)-based fluorescent probes for ONOO^-^

NPs refer to the specific materials of which at least one dimension lies in the range of 1-100 nm. Owing to their several unique features, such as enhanced permeability and retention effect, NPs have been widely used in the bioimaging field 111. However, the reports of nanoprobes for detection of ONOO^-^ are relatively few, which can be divided into two types: nonemissive NPs and fluorescent NPs. The former can serve as carriers for molecular fluorescent dyes, while the latter alone can give optical signals toward for certain analytes. Molecular dyes are assembled as fluorescent couples for multi-channel detection. Herein, we dissuss them according to diverse nano-platforms, including polymeric NPs, quantum dots (QDs), upconversion NPs (UCNPs), and other NPs.

### QDs

QDs are an important class of nanomaterials composed of semiconductors, which are usually spherical in shape. Owing to their small size, typically in the range of 2-10 nm, QDs can display quantum confinement effects, ultimately producing size-dependent tunable emission 112. On the other hand, the emission wavelength of QDs can be altered by the choice of their compositions.

Zhao *et al.* developed a type of ratiometric fluorescent nanoprobe for ONOO^-^, which was constructed by covalent coupling of graphene quantum dots (GQDs) with cyanine 5.5 (Cy5.5) 113. This nanoprobe (GQD-Cy5.5) could selectively accumulate in the mitochondria, giving rise to two strong fluorescence emission peaks at 520 and 694 nm. In the presence of ONOO^-^, the intensity of fluorescence emission peak at 520 nm increased, while the intensity of fluorescence emission peak at 694 nm decreased. The ratio of fluorescence intensity at two emission peaks (*F*_520 nm_/*F*_694 nm_) had a good linear relationship with the concentration of ONOO^-^ in the range of 0-6 μM. Furthermore, an apparent increase in the fluorescence was visualized in the nanoprobe-treated RAW264.7 cells stimulated by SIN-1, LPS, suggesting the ability of the nanoprobe to monitor exogenous and endogenous ONOO^-^ in living cells.

**Figure 20:**
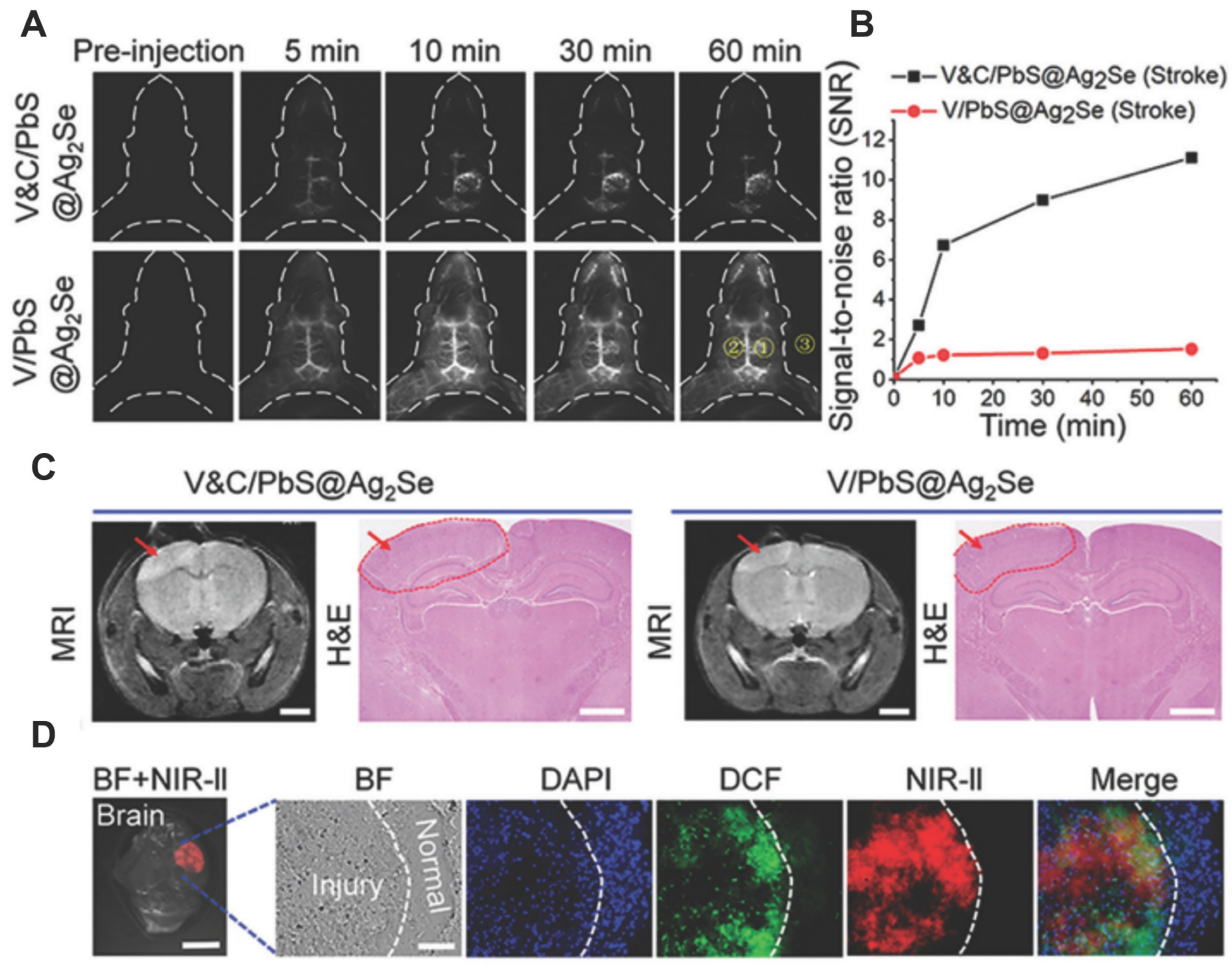
(A) Time course of NIR-II fluorescence in early ischemic stroke at different time points after injection of V&C/PbS@Ag_2_Se and V/PbS@Ag_2_Se. (B) Time-dependent SNR changes determined by the NIR-II fluorescence imaging of mice with different nanoprobes. SNR = [(mean fluorescence intensity of 1)-(mean fluorescence intensity of 3)]/[(mean fluorescence intensity of 2)-(mean fluorescence intensity of 3)]. (C) MRI and H&E staining detection of early ischemic stroke after illumination for 10 min. MRI scale bar: 2 mm, H&E scale bar:1 mm. (D) Fluorescence images of brain slice obtained from the excised brain tissue. Adapted from 115 with permission.

A notable shortcoming of the probes for ONOO^-^ detection described above is their short-wavelength emission, which hinders their application in bioimaging. Wang *et al.* used an activatable NIR-II nanoprobe was used for ONOO^-^ bioimaging 114. The nanoprobe V&A@Ag_2_S included three components: VCAM1 binding peptide (VHPKQHR), a NIR absorber A1094, and Ag_2_S QD, in which the Ag_2_S QD with emission at 1050 nm was employed as an energy donor and an ONOO^-^-responsive A1094 chromophore was used as the energy acceptor. Initially, the fluorescence of V&A@Ag_2_S displayed an“off”state owing to energy transfer from Ag_2_S to the A1094 chromophore. Upon intravenous injection, V&A@Ag_2_S quickly accumulated in the inflamed vascular endothelium of TBI based on VCAM1-mediated endocytosis, after which the nanoprobe achieved rapid recovery of the NIR-II fluorescence of Ag_2_S QDs owing to the bleaching of A1094 by the ONOO^-^. Taking advantage of the unique optical properties of NIR-II Ag_2_S QDs, the authors demonstrated the feasibility of such nanoprobes for in vivo ONOO^-^ detection in a traumatic brain injury (TBI) model. The same research group developed a NIR-II nanoprobe Cy7.5 fluorophore coordinate with PbS@Ag_2_Se QDs for ONOO^-^ using the same strategy 115. These results validated the capacity of the as-prepared V&C/PbS@Ag_2_Se nanoprobes for ONOO^-^ detection in early ischemic stroke (Figure 20).

### UCNPs

Although luminescent NPs, such as QDs, have achieved considerable development in the detection and bioimaging of ONOO^-^, their practical applications are somewhat limited by short excitation wavelengths, which are deemed unsuitable for deeper imaging in tissues or *in vivo*. One of the methods to overcome this issue is the exploration of UCNPs, which can convert long-wavelength excitation (generally in the NIR region) to short-wavelength emission. This is because with different lanthanide doping, UCNPs can obtain multiple emissions with 980 nm excitation 116. Thus, it can be used in the design and synthesis of ratiometric fluorescent probes for sensing and imaging *in vivo*.

In 2019, Yuan *et al*. developed two nanoprobes based on the energy transfer (ET) strategy. The UCNPs as donors modified with highly selective dye to ONOO^-^ as receptors through the polyethyleneimine (PEI) between the amino groups of PEI and carboxyl groups of the dyes 117. Upon being modified with dyes E-CC and H-CC, the upconversion luminescence (UCL) of UCNPs at 540 and 660 nm was quenched, respectively. Upon the nanoprobes (UCNPs@PEI@E-CC or UCNPs@PEI@H-CC) recognition for ONOO^-^, E-CC or H-CC would be destroyed, the ET was blocked, and the UCL at 540 or 660 nm was recovered. Thus, the ratio of intensities of UCNPs@PEI@E-CC at 540 and 660 nm (*I*_540_/*I*_660_) or ratio of UCNPs@PEI@H-CC at 660 and 810 nm (*I*_660_/*I*_810_) enabled the quantitative measurement of ONOO^-^. A good selectivity of nanoprobes toward ONOO^-^ over other species was further confirmed. Finally, the ONOO^-^ fluctuations in CCl_4_-induced hepatotoxicity in mice were successfully monitored using the nanoprobe.

Also based on the ET strategy, Yi *et al.* fabricated another nanoprobe for evaluating drug-induced hepatotoxicity by using heptamethine cyanine dye (P-cy7)-coordinated UCNPs 118. Under the excitation of a 980 nm laser, the “off-on” luminescence at 656 nm and always “on” luminescence at 800 nm of UCNPs@P-cy7 were used as ratio-fluorescent nanoprobes for the quantitative detection and ratio-fluorescent imaging of ONOO^-^. Furthermore, the authors noted that the absorption change in the nanoprobes in the presence of ONOO^-^ could lead to a change in the PA signals to achieve highly sensitive PA imaging. The authors discovered using dual-mode ratio-fluorescent imaging and PA imaging that the nanoprobes were applicable for accurately diagnosing different damage degrees of hepatotoxicity.

### Other NPs

Zhou *et al.* developed a two-photon NIR ratiometric nanoprobe NTC for ONOO^-^, which was constructed by self-assembling a small-molecule fluorescent probe (NR) grafted onto azide chitosan (natural polymeric nanomaterial) 119. The free NTC exhibited a strong fluorescence intensity at 700 nm and a weaker characteristic band at 526 nm. In the presence of ONOO^-^, the fluorescence intensities at 700 and 526 nm gradually decreased and increased, respectively. In addition, NTC exhibited a short response time (~10 s) and high selectivity and sensitivity toward ONOO^-^, with an excellent LOD as low as 15.3 nM. Notably, NTC was successfully employed for ONOO^-^ detection and imaging in living HepG2 cells, liver injury mice tissues, and mice models.

Another type of NPs, an energy transfer scaffold, was constructed by conjugation of a NIR xanthane fluorophore with a rhodamine B fluorophore, which was then grafted onto sodium chondroitin sulfate (CSNa) to form CSU-FT through self-assembly 120. The nanoprobe exhibited bright emission at 707 nm. After the addition of ONOO^-^, the fluorescence intensity of the nanoprobe increased significantly at 468 and 573 nm and decreased sharply at 706 nm. A good linearity was found between the fluorescence intensity ratios (*I*_468_/*I*_573_, *I*_468_/*I*_706_, and *I*_573_/*I*_706_) and the ONOO^-^ concentration in the range of 0-20 μM, and the LOD was calculated to be 11.7 nM. Finally, CSU-FT was successfully applied for the detection of ONOO^-^ in RAW 264.7 cells and rat, and its diagnostic and therapeutic efficiency for arthritis was demonstrated in rat experiments.

By coating with silica nanoparticle the Tb(DPA)_3_ as reference dye and attaching the responsive dye (TD) to the surface, Liu *et al.* fabricated a nanoprobe for ONOO^-^ detection 121. The hydrodynamic diameter of the spherical nanoprobe was measured as 30 nm. With the addition of ONOO^-^ (0-12 μM), the emission decreased apparently at 620 nm and enhanced distinctly at 547 nm. In addition, the response time was also considered a vital indicator for real-time detection. Subsequently, the ONOO^-^ in A-375 cells was visualized, indicating the ability of the nanoprobe to sense the ONOO^-^.

A novel ONOO^-^ and environmental pH dual-responsive afterglow luminescent nanoprobe TPE-TV-CyP, synthesized by Ding's group, exhibited luminescence depends on the integrated influence of ONOO^-^ and pH dual-stimuli 122. The probe selectively reacted with ONOO^-^ to afford a 553-fold fluorescence increase. Detection of ONOO^-^ further reveal the development process of acute skin inflammation including infiltration of first arrived neutrophils and acidification initiating time, make a fast and accurate discrimination between allergy and inflammation, and rapidly screen the antitumor drugs capable of inducing immunogenic cell death. The same group designed another nanoprobe Ppa-FFGYSA based upon successive oxidation of vinylene 123. After addition of ONOO^-^ resulted in an intensity enhancement at 760 nm and decay half-lives of approximately 2-15 min. Interestingly, the novel probe could transform light-triggered function from photoacoustic imaging to persistent luminescence imaging permit advanced image-guided cancer surgery.

## Conclusions and outlooks

In summary, we systemically introduce fluorescence probes for ONOO^-^ imaging in the last 5 years. We have classified the enumerated probes based on six different reaction mechanism, emphasize their response type (ratiometric type, two-photon type, long-wavelength/NIR type), and elaborated on their reaction mechanisms as well as bioimaging applications. Further, we also describe nanoparticle (NP)-based probes according to diverse nanoplatforms. In particular, details of their mechanism, response type, emission wavelengths, detection limits, detailed probe's bio-applications are listed in tables (Table 1-8).

According to Table 1-8, probes with NIR-II are really poorly, which limits their bio-imaging application. In addition, the ONOO^-^ probes constructed from responsive mechanism, such as oxidation of boronates, C=C cleavage mechanism, N-dearylation mechanism, have proven more profound in disease models. In contrast, probes constructed from hydrazide, ketones, and chalcogenides are relatively poorly applied in tissue imaging and disease models. Most probes responded to ONOO^-^ within seconds and the LOD reaches nM. Due to the ONOO^-^ chemical properties, low content (nM-µM) and short life (~ms) in the complicated biological environment, it is urgent to design “perfect” sensitivity and selectivity probes. In the meantime, the selectivity of some ONOO^-^ probes needs to be further improved. We hope that this review would help the researchers interested in discovering new probes for detecting ONOO^-^ or novel reaction mechanisms differing from the traditional ones.

The current reaction mechanism is primarily the oxidation reaction including oxidation of boronic acids and its ester derivatives, oxidation-induced cleavage of ethylenic bond, oxidation of α-ketoamide derivatives, and so on, which means simple sensing mechanism and probes may interfere with other active species such as H_2_O_2_. Based on the current research status of ONOO^-^ probes, in the next few years, the following (non-exhaustive) ways might be valuable: (1) The priority of research should be ''back to basic” to explore unique and creative reaction modes based on the deep exploration of chemical-physical properties of ONOO^-^. (2) The function of ONOO^-^ in other organelles besides mitochondria such as the endoplasmic reticulum need to be explored. Additionally, more pathologies associated with ONOO^-^ will be certainly uncovered. (3) Some factors should be considered in the design of probes for biological systems, such as good solubility, good cell permeability, and low interference from biological environments. NIR and two-photon probes have great potential for bioimaging of ONOO^-^ because of their properties of low background interference and high penetrability of low energy light into tissues. (4) The diagnosis and treatment function of the probe should be further explored. After the probe reacts with ONOO^-^, the concentration of ONOO^-^ is reduced to a certain extent. In other words, the probe acts as an ONOO^-^ scavenger. Therefore, how to use ONOO^-^ probes to realize the integration of diagnosis and treatment may be considered another important research-direction in the future. (5) To solve the difficulty for the imaging changes in the sub-organelles, researchers can combine the advanced super-resolution fluorescence microscopy techniques with fluorescent probes. This is anticipated to provide more precise information concerning ONOO^-^ variation in sub-organelles for better understanding of related physiological or pathological processes.

On the whole, we anticipate that this review will provide a comprehensive knowledge of the design of fluorescent probes for ONOO^-^ and their applications in bioimaging, and hope that the fluorometric imaging technique will offer more assistance in biomedical and clinical fields in the near future.

## Figures and Tables

**Table 1:** Small-molecule fluorescent probes based on boronates (boronic acids/boronic esters).

Sensor	λ_em_ (nm)	Response type	Response time	LOD	Biological applications	Refs
**1**	610	Reaction-based indicator displacement assay (RIA)	< 40 s	—	Exogenous ONOO^-^ imaging in HepG-2 cells.	23
**2**	494	Reaction-based indicator displacement assay (RIA)	< 2 s	3.64 nM	ONOO^-^ imaging in HepG-2, HL7702 and HeLa cell lines.	24
**4**	530	-	< 20 min	15 nM	ONOO^-^ imaging in HepG-2 cells, in drug-damaged liver tissues.	26
**5**	385 450	Ratiometric	5 min	29.8 nM	ONOO^-^ imaging in RAW264.7 cells, EAhy926 cells, zebrafish and live tissues from a high-fat diet-induced obese mouse model.	28
**6**	405 481	Ratiometric Mitochondria-targeting Two-photon imaging	—	—	ONOO^-^ imaging in RAW 264.7 cells	29
**7a**	447	Ratiometric Mitochondria-targeting	—	0.28 µM	ONOO^-^ imaging in RAW 264.7 macrophages.	30
**8**	405 481	Ratiometric Endoplasmic reticulum-targeting	—	21.4 nM	ONOO^-^ imaging in HeLa cells; colocalization experiments;	31
**9**	450 540	Ratiometric	< 30 s	0.97 µM	Exogenous ONOO^-^ imaging in HepG-2 cells; endogenous ONOO^-^ in RAW 264.7; in situ image ONOO^-^ in the zebrafish.	32
**10**	560 630	Ratiometric Far-red-emitting	< 5 s	0.9 nM	Endogenous ONOO^-^ imaging in RAW 264.7 macrophage cells	33
**11a**	544 655	Ratiometric Lysosomes-targeting	< 5 min	8.4 nM	ONOO^-^ imaging in HepG-2 cells; colocalization experiments; ONOO^-^ imaging in APAP-induced hepatotoxicity and GSH remediation in HepG-2 cells; in LPS-induced nude mice arthritis models; in APAP-induced liver injury model in mice.	34
**12**	510 625	Ratiometric Mitochondria-targeting	70 min	0.45 nM	Colocalization experiments; ONOO^-^ imaging in RAW264.7 cells.	35
**13**	569	Mitochondria-targeting	< 1 min	16 nM	Colocalization experiments; endogenous ONOO^-^ imaging in HeLa cells; H_2_S could scavenge endogenous ONOO^-^.	39
**14**	451	'AND' logic gate	—	—	GSH and ONOO^-^ imaging in RAW264.7 cells	40
**15**	512	'AND' logic gate	—	—	GSH and ONOO^-^ imaging in RAW264.7 cells	41
**16**	450 543	Ratiometric Two-photon imaging	< 10 s	1.4 nM	Two-photon ONOO^-^ imaging in RAW 264.7 macrophage cells	44
**17**	550	Two-photon imaging N-methyl-D-aspartate (NMDA)-targeting	—	184 nM	One-photon ONOO^-^ imaging in live neuroblastoma cells (SH-SY5Y); two-photon ONOO^-^ imaging in primary cortical neuronal cells and rat hippocampal slice	45
**18**	515	Two-photon imaging	—	73 nM	Two-photon ONOO^-^ imaging in HeLa cells; rat hippocampal slices	46
**19**	520	Two-photon imaging	—	—	Two-photon ONOO^-^ imaging in RAW264.7 cells	47
**20**	606	Mitochondria-targeting Long-wavelength-emitting	—	—	ONOO^-^ imaging in HepG-2, RAW 264.7, HeLa, and A459 cell lines.	48
**21**	620	Long-wavelength-emitting	< 4 min	27.5 nM	ONOO^-^ imaging in EC1 cells.	49
**22**	625	Long-wavelength-emitting	**—**	19.8 nM	ONOO^-^ imaging in RAW 264.7 cells	50
**23**	720	NIR -emitting	**—**	3.54 µM	ONOO^-^ imaging in MCF-7 cells.	51
**24**	657	NIR -emitting	< 30 s	50 nM	ONOO^-^ imaging in MCF-7 cells and RAW 264.7 macrophage cells; in LPS-induced leg inflammation of the mouse model; in LPS-induced peritonitis mouse model;	52
**25**	692	NIR -emitting	< 15 min	94 nM	Endogenous ONOO^-^ in living human neuroblastoma SH-SY5Y cells; in epilepsy mouse model.	53
**27**	635	Long-wavelength-emitting	< 5 min	1.69 nM	ONOO^-^ imaging in HeLa cells; endogenous ONOO^-^ imaging in zebrafish; exogenous ONOO^-^ imaging in mice; ONOO^-^ and viscosity imaging in APAP-induced hepatotoxicity in livers of mice.	55
**28**	850	NIR-emitting	< 3 min	55.9 nM	ONOO^-^ imaging in APAP-induced hepatotoxicity in nude mice models.	56

**Table 2:** Small-molecule fluorescent probes based on C=C/C=N.

Sensor	λ_em_ (nm)	Response type	Response time	LOD	Biological applications	Refs
**29**	456	Lipid droplet-targeting	< 3 min	326 nM	Co-localization experiments; ONOO^-^ imaging in HepG-2 cells; ONOO^-^ imaging in cyclophosphamide (CP)-induced living cells; evaluated ONOO^-^ scavengers N-acetyl cysteine (NAC)/Glutathione repair the damage caused by CP.	57
**30**	520	Mitochondria-targeting	**—**	0.21 μM	ONOO^-^ imaging in HepG-2 cells; colocalization experiments	58
**31**	510	Lysosome-targeting	< 10 s	0.24 µM	ONOO^-^ and viscosity imaging in RAW264.7 cells; colocalization experiments	59
**32**	650	NIR-emitting	1 min	26 nM	ONOO^-^ imaging in RAW264.7 cells;	60
**33**	487 742	Ratiometric Mitochondria-targeting	150 s	0.17 µM	ONOO^-^ imaging in HepG-2 cells and mice; colocalization experiments	61
**34**	461	Two-photon imaging	**—**	38.2 nM	ONOO^-^ and O_2_^.-^ imaging in HL-7702 cells and mice modes;	62
**35**	485 650	Ratiometric Mitochondria-targeting	**—**	12.1 nM	Co-localization experiments; ONOO^-^ imaging of the mitochondrial in HepG-2 cells and RAW macrophages; in rat liver slice.	63
**36**	510 606	Ratiometric	< 1 min	150.54 nM	ONOO^-^ imaging in RAW264.7 cells, THP-1 cell line and LPS-induced mouse inflammation model.	64
**37**	564 700	Ratiometric Mitochondria-targeting NIR-emitting	10 s	28.06 nM	ONOO^-^ imaging in RAW264.7 cells; in an acute inflammation mice model; in the rheumatoid arthritis model.	65
**38**	535 718	Ratiometric Mitochondria-targeting NIR-emitting Two-photon imaging	**—**	85 nM	Co-localization experiments; ONOO^-^ imaging in A549 cells and RAW 264.7 cells; Bleomycin-induced pulmonary fibrosis and remediation effect of Ag;	66
**39**	680 750	Mitochondria-targeting NIR-emitting Ratiometric	**—**	0.36 µM	ONOO^-^ imaging in cisplatin-caused nephrotoxicity HK-2 cells and mice models;	67
**40**	524	-	< 60 s	58 nM	ONOO^-^ imaging in Hela cells.	68
**41**	525	-	< 15 s	85.7 nM	Super-resolution visualization of the ONOO^-^ imaging in HeLa cells;	69

**Table 3:** Small-molecule fluorescent probes based on hydrazides.

Sensor	λ_em_ (nm)	Response type	Response time	LOD	Biological applications	Refs
**42**	638	NIR-emitting	**—**	45 nM	Exogenous ONOO^-^ imaging in HeLa cells; endogenous ONOO^-^ imaging in RAW 264.7 cells; endogenous ONOO^-^ in PAO1-infected mouse bone marrow-derived neutrophils.	70
**43**	660	NIR-emitting Two-photon imaging	**—**	15 nM	Exogenous ONOO^-^ imaging in HeLa cells; endogenous ONOO^-^ imaging in RAW264.7 cells; in mouse liver tissue; in inflammatory mouse models; in APAP-induced hepatotoxicity in HepG-2 cells.	71
**44**	630	Mitochondria-targeting NIR-emitting	< 5 s	17 nM	Co-experiments; ONOO^-^ imaging in HeLa cells.	72
**45**	698	NIR-emitting	< 2 s	25 nM	Exogenous ONOO^-^ imaging in HeLa cells; endogenous ONOO^-^ imaging in RAW264.7 cells.	73
**46**	585	-	< 3 s	0.68 nM	ONOO^-^ imaging in RAW 264.7 macrophages; in zebrafish.	74
**47**	575	Sodium-dependent multivitamin transporter (SMVT)-targetable	< 1 min	7 nM	Endogenous ONOO^-^ imaging in Cal-27 cells; targeting experiment in HSC-2 cells; in tumor-derived nude mice.	75
**48**	500	-	**—**	22.6 nM	ONOO^-^ imaging in LM-3 cells; in BALB/c mice.	76
**49**	515 700	Ratiometric NIR-emitting	**—**	59 nM	Exogenous ONOO^-^ imaging in HeLa cells; endogenous ONOO^-^ imaging in RAW264.7 cells; in living mice models.	77
**50**	505 578	Lysosome-targeting Ratiometric Two-photon imaging	< 10 s	3.33 nM	Co-localization experiment; ONOO^-^ imaging in HeLa cells, rat liver tissues and zebrafish.	78
**51**	474 574	Ratiometric Two-photon imaging	< 2 min	**—**	ONOO^-^ imaging in RAW 264.7 cells; in murine macrophages in atherosclerotic plaques; revealed the ONOO^-^ content of macrophages is inversely related to arginase 1 activity.	79

**Table 4:** Small-molecule fluorescent probes based on N-dearylation.

Sensor	λ_em_ (nm)	Response type	Response time	LOD	Biological applications	Refs
**52**	500	Two-photon imaging	< 1 min	35 nM	Endogenous ONOO^-^ imaging in RAW 264.7 cell; in rat hippocampal slices.	80
**53**	585	-	Within seconds	**—**	ONOO^-^ imaging in EA.hy926 endothelial cells.	81
**54**	540	Two-photon imaging Endoplasmic reticulum -targeting	< 1 s	8.3 nM	ONOO^-^ imaging in PC12 cells and PD models in WLZ3 C. *elegans*.	82
**55**	670	NIR-emitting	1 s	4.59 nM	ONOO^-^ imaging in PC12 cells and SHSY5Y cell line; in multiple PD models including PC12 cell, Drosophila, *C. elegans*, and mouse brain.	83
**56**	560	Near-infrared-emitting	< 25 s	0.13 µM	ONOO^-^ imaging in Human hepatic stellate LX-2 cells; in acute liver injury (ALI) model mice.	84
**57**	679	NIR-emitting kidney-targeting	Within seconds	100 nM	ONOO^-^ imaging in HK-2 cells; in RAW 246.7 cells after injury induced by H_2_O_2_ and CIS; in CIS- or IR-induced AKI live mice.	85

**Table 5:** Small-molecule fluorescent probes based on ketones.

Sensor	λ_em_ (nm)	Response type	Response time	LOD	Biological applications	Refs
**58**	630	Two-photon imaging NIR-emitting Mitochondria-targeting	10 s	34 nM	Colocalization experiment; ONOO^-^ imaging in anthracycline cardiotoxicity H9c2 cardiomyocyte model; in related cardiotoxicity in mouse models.	86
**59**	630	NIR-emitting	< 90 s	78 nM	Endogenous ONOO^-^ imaging in macrophage J774A.1 cells	87
**60**	703	NIR-emitting	**—**	90 nM	ONOO^-^ imaging in HepG-2 cells; in DILI Model; the therapy of hepatoprotective medicine after APAP-induced hepatotoxicity in HepG-2 cells.	88
**61**	712	NIR-emitting	**—**	53 nM	ONOO^-^ imaging in RAW264.7 cells; NIRF and PA imaging of ONOO^-^ in the subcutaneous 4T1 xenograft tumor of living mice.	89
**62**	717	NIR-emitting	< 2 min	89.4 nM	ONOO^-^ imaging in living cells; in vivo fluorescence imaging of keloid.	90

**Table 6:** Small-molecule fluorescent probes based on chalcogenides.

Sensor	λ_em_ (nm)	Response type	Response time	LOD	Biological applications	Refs
**63**	512	-	< 10 min	0.4 µM	ONOO^-^ imaging in RAW 264.7 macrophages.	91
**64**	480 630	Ratiometric	9 min	19.4 nM	ONOO^-^ imaging in MCF-7 cells; in 5-FU-treated EC1 cells.	92

**Table 7:** Small-molecule fluorescent probes based on other mechanisms.

Sensor	λ_em_ (nm)	Response type	Response time	LOD	Biological applications	Refs
**65**	550	Two-photon imaging	6 min	16 nM	One and two-photon fluorescence imaging of ONOO^-^ in RAW264.7 cells.	93
**66**	555	Hepatoma-targeting	< 3 s	20 nM	ONOO^-^ imaging in HepG2 cells.	94
**67**	553	Two-photon imaging	< 30 min	6.2 nM	Exogenous ONOO^-^ imaging in SMMC-7721 cells; peroxisomal targeting; in the livers of mice during ALI.	95
**68**	473 651	Ratiometric Mitochondria-targeting Two-photon imaging	< 20s	11.3 nM	Endogenous ONOO^-^ imaging in HepG-2 and RAW264.7 cells; two-photon living hepatic tissue imaging of endogenous ONOO^-^ and in the inflamed living mouse model.	96
**69**	469 703	Ratiometric Two-photon imaging	**—**	4.1 nM	Co-localization experiments; ONOO^-^ imaging in nonalcoholic fatty liver (NAFLD) with drug-induced liver injury (DILI) diseases in L02 cells; in the high-fat-diet-caused mouse model of NAFLD.	97
**70**	462 626	Ratiometric	< 30min	1.8 nM	ONOO^-^ imaging in RAW264.7 Cells; in the ALI model in L02 cells.	98
**71**	500 720	Ratiometric Hepatoma-targeting NIR-emitting	**—**	0.17 mM	ONOO^-^ imaging in HepG-2 cells; in DILI and GSH、NAC and DDB remediation in BALB/c mice.	99
**72**	525 606	Long-wavelength-emitting	< 1 s	1.2 nM	ONOO^-^ imaging in RAW 264.7 cells.	100
**73**	506	Two-photon imaging	< 10 s	**—**	Two-photon fluorescence imaging of ONOO^-^ (green channel) and Aβ aggregates (blue channel) in PC12 cells and in brain tissues of AD mouse models.	101
**74**	557	NIR-emitting	< 120 s	**—**	TP imaging of ONOO^-^ in living RAW 264.7 cells; in LPS-induced kidney injury of zebrafish; in mouse brain with stroke.	102
**75**	535 628	Ratiometric NIR-emitting	< 9min	13.3 nM	ONOO^-^ imaging in HeLa cells; co-localization experiments; in zebrafish.	103
**76**	525 632	Ratiometric Aggregation-induced emission luminogens (AIEgens)	**—**	30 nM	ONOO^-^ imaging in HeLa cells; in live mice.	104
**77**	685	NIR-emitting Two-photon imaging	< 10min	96 nM	ONOO^-^ imaging in RAW 264.7 cells; in living HT22 cells; in rat epilepsy models.	105
**78**	680	NIR-emitting	< 20 s	3 nM	Exogenous ONOO^-^ imaging in HepG-2 cells; endogenous ONOO^-^ imaging in RAW264.7 macrophages; endogenous ONOO^-^ in endothelial cells during IR injury;	106
**79**	599	-	5 s	13 nM	ONOO^-^ imaging in SMMC-7721 cells.	107
**80**	495	-	< 40s	19 nM	ONOO^-^ imaging in RAW 264.7 cells; endogenously induced by APAP and heat shock.	108
**81**	525	-	2 s	90 nM	Endogenous ONOO^-^ imaging in H9C2 cells;	109
**82**	454 558	Ratiometric Mitochondria-targeting	< 20min	0.12 µM	Co-localization experiments; endogenous ONOO^-^ imaging in HeLa and RAW264.7 cells.	110

**Table 8:** Nanoparticle (NP)-based fluorescent probes for ONOO^-^.

Nanoprobe	Size	Response type	λ_em_ (nm)	LOD	Biological applications	Refs
GQD-Cy5.5	3.5 nm	Ratiometric Mitochondria-targeting	520 694	0.03 μM	Exogenous and endogenous ONOO^-^ imaging in RAW264.7 macrophages;	113
V&A@Ag_2_S	3.3 nm	NIR-II	1050	0.06 μM	Endogenous ONOO^-^ in HUVECs cells; ONOO^-^ imaging in brain vascular injury;	114
V&C/PbS@Ag_2_Se	150 nm	NIR-II	1600	-	Endogenous ONOO^-^ in HUVECs Cells; ONOO^-^ imaging in early ischemic stroke injury; biotoxicity assessment.	115
UCNPs@PEI@E-CC	28 nm	Ratiometric	540 810	154 nM	Exogenous and endogenous ONOO^-^ imaging in HepG-2 cells;	117
UCNPs@PEI@H-CC	28 nm	Ratiometric	660 810	241 nM	-	117
UCNPs@P-cy7	27-35 nm	Ratiometric	656 800	-	ONOO^-^ imaging in vitro RFLI and PAI. In vivo RFLI and PAI of endogenous ONOO^-^ in the livers of mice during APAP-induced hepatotoxicity;	118
NTC	80 nm	Ratiometric NIR-emitting Two-photon imaging	526 700	15.3 nM	ONOO^-^ imaging in live HepG2 cells, liver injury mice tissues, and mice models.	119
CSU-FT	25.9 nm	Ratiometric	468 573 706	11.7 nM	Exogenous and endogenous ONOO^-^ imaging in RAW264.7 macrophages; ONOO^-^ imaging in living muscle tissue and inflamed living rat model; diagnosis and therapy of arthritis mice.	120
TD-DC	30 nm	Ratiometric Mitochondria-targeting	547 620	39 nM	ONOO^-^ imaging in A-375 cells.	121
TPE-TV-CyP	110 nm	Afterglow luminescence	640	34 nM	ONOO^-^ imaging in neutrophils infiltration and acidification process of acute inflammation; in inflammatory and allergic diseases models.	122
Ppa-FFGYSA	-	Persistent luminescence	760	64.2 nM	Persistent luminescence in chicken tissues; PA imaging in 4T1 tumor-bearing mice; imaging in photodynamic therapy of hypericin to induce strong immunogenic cell death of tumor cells;	123
